# Combined genotyping, microbial diversity and metabolite profiling studies on farmed *Mytilus* spp. from Kiel Fjord

**DOI:** 10.1038/s41598-018-26177-y

**Published:** 2018-05-22

**Authors:** Caroline Utermann, Delphine Parrot, Corinna Breusing, Heiko Stuckas, Tim Staufenberger, Martina Blümel, Antje Labes, Deniz Tasdemir

**Affiliations:** 1 GEOMAR Centre for Marine Biotechnology (GEOMAR-Biotech), Research Unit Marine Natural Products Chemistry, GEOMAR Helmholtz Centre for Ocean Research Kiel, Am Kiel-Kanal 44, 24106 Kiel, Germany; 2 Research Unit Evolutionary Ecology of Marine Fishes, GEOMAR Helmholtz Centre for Ocean Research Kiel, Duesternbrooker Weg 20, 24105 Kiel, Germany; 3 Senckenberg Natural History Collection Dresden, Population Genetics, Koenigsbruecker Landstrasse 159, 01109 Dresden, Germany; 4 Kieler Meeresfarm GmbH, Richthofenstrasse 31, 24159 Kiel, Germany; 5 Present Address: Monterey Bay Aquarium Research Institute, 7700 Sandholdt Road, Moss Landing, California, 95039 USA; 6 Present Address: Flensburg University of Applied Sciences, Kanzleistrasse 91-93, 24943 Flensburg, Germany; 7 Kiel University, Christian-Albrechts-Platz 4, 24118 Kiel, Germany

## Abstract

The blue mussel *Mytilus* is a popular food source with high economical value. Species of the *M*. *edulis* complex (*M*. *edulis*, *M*. *galloprovincialis* and *M*. *trossulus*) hybridise whenever their geographic ranges overlap posing difficulties to species discrimination, which is important for blue mussel aquaculture. The aim of this study was to determine the genetic structure of farmed blue mussels in Kiel Fjord. Microbial and metabolic profile patterns were studied to investigate a possible dependency on the genotype of the bivalves. Genotyping confirmed the complex genetic structure of the Baltic Sea hybrid zone and revealed an unexpected dominance of *M*. *trossulus* alleles being in contrast to the predominance of *M. edulis* alleles described for wild Baltic blue mussels. Culture-dependent and -independent microbial community analyses indicated the presence of a diverse *Mytilus*-associated microbiota, while an LC-MS/MS-based metabolome study identified 76 major compounds dominated by pigments, alkaloids and polyketides in the whole tissue extracts. Analysis of mussel microbiota and metabolome did not indicate genotypic dependence, but demonstrated high intraspecific variability of farmed mussel individuals. We hypothesise that individual differences in microbial and metabolite patterns may be caused by high individual plasticity and might be enhanced by *e.g*. nutritional condition, age and gender.

## Introduction

The blue mussel, a filter-feeding mollusk belonging to the colony forming bivalve genus *Mytilus*, is common in many marine environments. This mollusk is considered as an important ecosystem engineer due to its contribution to habitat biodiversity and structure within coastal and estuarine communities^1^. Environmental monitoring programs frequently use blue mussels for assessment of water quality with respect to ecotoxic compounds^2^. Blue mussels represent one of the major aquaculture shellfish species. In many countries, they constitute one of the most valuable food-based primary industries besides beef, wheat and dairy^3^. Several constituents of *Mytilus* spp. are valuable for public health such as the anti-inflammatory omega-3 polyunsaturated fatty acids eicosapentaenoic acid and docosapentaenoic acid^4,5^. The identity of blue mussel populations is therefore important for the farmer to sell certified products and guarantee correct provenance determination to the customer. Morphological features, mainly shell characteristics, have been used for a long time in species discrimination of blue mussels^6^. However, high phenotypic plasticity, particularly variations of shell morphology by environmental influences (*e*.*g*. wave exposure and salinity), diminishes interspecific differences and renders morphology unreliable for species discrimination^7^. Wherever their geographical range overlaps, different *Mytilus* spp. hybridise to form genetically complex assemblages and hybrid zones of different extents^8^. Accordingly, the taxonomy of *Mytilus* spp. proves very difficult to resolve. Therefore, species discrimination is nowadays based on genetic features, including nuclear (*e*.*g*. EFbis, Glu-5′, ITS^7^), mitochondrial (*e*.*g*. D-loop^9^), allozyme (*e*.*g*. Gpi, Pgm^7^) and SNP markers^10–13^. The use of SNP markers recently brought advances in delimiting species status of wild populations and contributed to the understanding of the extent of interspecific gene flow^14^. Currently, three *Mytilus* species, namely *M*. *edulis* (*Me*), *M*. *galloprovincialis* (*Mg*) and *M*. *trossulus* (*Mt*) are regarded as distinct species, forming the *Me* complex^6^. *Mg* and *Mt* hybridise in the NW Pacific and NE Pacific^15^. *Me* interbreeds with *Mg* in the NE Atlantic and with *Mt* in the NW Atlantic and the Baltic Sea^15^. In those hybrid zones, interbreeding and retention of pure species genomes are regulated by genetic reproduction barriers^16,17^. Strong reproductive isolation is also seen in the *Me* x *Mt* hybrid zones in the North and Norwegian Seas^16^. Reproductive isolation is caused by a variety of mechanisms in marine systems, such as differences in spawning time, mate recognition, environmental tolerance, and gamete compatibility, maintaining genetic divergence^18^. In contrast, in the Baltic Sea *Me* and *Mt* populations face a low reproductive isolation facilitating interbreeding of the two species^16,17^. The Baltic Sea encounters a strong genetic shift along a virtual line from Malmö (Sweden) to Stralsund (Germany)^19,20^. *Mt* alleles dominate in the inner Baltic Sea (east of the virtual line) while *Me* alleles are more pronounced on the west side of the transition zone^16,17,20^. Until now, no information is available regarding the degree of introgression in farmed mussels on long lines in the same area.

The emerging ‘metaorganismal view’ of macroorganisms describes the microbiota – the associated microbial community of a macroorganism host – as part of an individual’s phenotype^21^. The microbiota are crucial for the host’s physiological and ecological performance and often produce bioactive compounds promoting the host’s immune system, reproduction, nutrition or defense^22,23^. As an example, symbiotic sponge microbes assimilate ammonia, the metabolic sponge waste-product and complement the sponge diet by producing vitamins^24,25^. The symbiotic cyanobacterium *Prochloron didemni* supplies its tunicate host (*Lissoclinum patella*) with fixed carbon, recycled nutrients and potential defensive metabolites such as the polyketide patellazole^26,27^. Compared to the extensive studies on *Mytilus* spp. itself, only a few studies have investigated *Mytilus*-associated microbiota. Most of these focus on toxigenic or bioactive properties of the microorganisms (*e*.*g*.^28,29^) or the cultivable microbial diversity (*e*.*g*.^30,31^). The microbiota associated with a host is generally considered being species-specific^32^. Only the recent advances in sequencing technologies allow us to study the applicability of the microbial diversity pattern as a species marker to support genotyping^33^. On the other hand, the associated microbial community of individuals of the same species often shows notable variation^34,35^. Hence, analysis of the intraspecific differences in the microbiota is of importance to fully encompass the range of genetic and metabolic diversity of the target organism^36^.

Metabolomics is directed towards comprehensive analysis of generally low molecular weight metabolites (<1000 Da) that are final products of many biological pathways essential for *i*.*e*. competitiveness and defense of any organism in its habitat^37^. Being closest to the phenotype, metabolomics is the ‘final’ tool in the ‘omics’ chain^38^. Metabolomics has a broad range of applications in ecology, *e*.*g*. for identification of spatial or seasonal variations or for evaluation of the impact of multiple stressors^39^. Marine pollution monitoring (*e*.*g*. heavy metals^40^) has been the main application of mussel metabolomics^2^. Metabolite fingerprinting, another application of metabolomics, is widely used as a tool for species discrimination of both terrestrial and marine organisms^41,42^. Recently, NMR and GC-MS based metabolomics have been successfully used for species discrimination and provenance determination of Australian blue mussels (*Mg*) and New Zealand green lipped mussels (*Perna canaliculus*)^43^. Thus, blue mussel metabolomics can assist genetic identification and provenance determination^43^. For *Mytilus* spp. it remains unknown whether different species or genetically distinct hybrids possess species-specific or genotype-independent microbial and metabolite profiles.

It has been reported that wild mussel populations in Kiel Fjord (KF) show striking morphological similarities^16^ and are dominated by *Me* alleles^19^. Nevertheless, some phenotypic traits of blue mussels, such as size, shell fragility and growth rate, have already been shown to be related to the genotype^7,44^. The aim of this study was to genotype individual farmed blue mussels with molecular markers. Next, we explored the microbiota of the same individuals by using both culture-dependent and -independent techniques to assess possible correlation or dependence between the microbial profile and the genotype. Using a rapid UPLC-QTOF-MS/MS protocol, we also analysed the blue mussel metabolome in order to detect a genotype-dependent chemical profile or a marker compound specific at species and/or hybrid level. Potential markers identified by microbial or metabolic fingerprinting may serve as fast and cheap genotyping tools that are useful for mussel farms. This is the first report on the metabolic and microbial composition of farmed Baltic *Mytilus* spp. and the first study combining genotypic, microbiology and metabolomics approaches on *Mytilus* species.

## Results

### Genotyping of *Mytilus* spp. individuals

**Figure 1:**
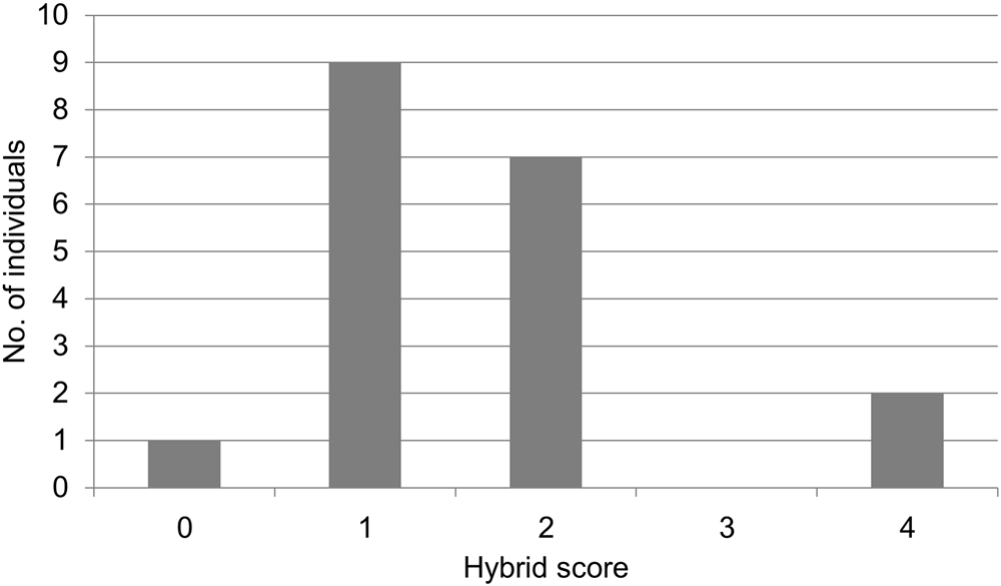
Genotypic distribution of cultured KF blue mussels. Genotypic assessment of 19 individuals was done by amplification with the nuclear markers EFbis and Glu-5′. Hybrid scores were calculated by counting all amplified alleles belonging to *Me*. A hybrid score of 4 is representing pure *Me* background (0: pure *Mt*, 1: *Mt*-like hybrid, 2: intermediate hybrid, 3: *Me*-like hybrid, 4: pure *Me*).

Genotyping of blue mussels was carried out by natural PCR product length variation with the two well-established nuclear markers EFbis and Glu-5′. Genetic classification of the 19 mussel specimens was carried out by the calculation of hybrid scores (HS) on the basis of reference samples (Fig. 1, see also Supplementary Table S1). Four different genotypes were observed, as *Me*-like hybrids (HS3) were not present. By accounting for nearly half of the sampled population (47%), *Mt*-like hybrids (HS1) clearly dominated. Another third of the individuals was assigned to the intermediate hybrid type (HS2) by carrying equal proportions of *Me* and *Mt* alleles. HS0 and HS4, reflecting the presence of pure *Mt* or *Me* without any genetic evidence for hybridisation, were detected either once (individual 19) or twice (individuals 5, 14) (Fig. 1), respectively.

Detailed allele frequency analysis of the two applied markers showed complete homozygosity for EFbis with an allele frequency of 0.84 for *Mt* alleles and 0.16 for *Me* alleles (Table 1, see also Supplementary Table S1). Equal proportions of heterozygous and homozygous individuals were obtained by Glu-5′ genotyping, resulting in allele frequencies of 0.29 for *Mt* and 0.71 for *Me*. Summarising the results of genotyping with the two markers, hybrid scores as well as allele frequencies indicate a dominance of *Mt* alleles over *Me* alleles in farmed blue mussels in KF (Fig. 1 and Table 1).

**Table 1:** Allele frequencies of two genes studied in blue mussels from KF.

Location	N	Glu-5′				EFbis		
		E/E	E/T	T/T	E/G	E/E	E/T	T/T
Blue mussel farm KF (this study)	19	0.47	0.47	0.05	0.0	0.16	0.0	0.84
KF: GEOMAR^33^	19	0.68	0.32	0.0	0.0	0.05	0.42	0.53
KF: Ship museum^33^	19	0.74	0.16	0.0	0.1	0.0	0.26	0.74
KF: Hörn^33^	19	0.90	0.05	0.0	0.05	0.05	0.32	0.63
KF: East shore^33^	20	0.80	0.20	0.0	0.0	0.0	0.40	0.60

M: *Me* allele, T: *Mt* allele, G: *Mg* allele, N: number of genotyped individuals.

### Profiling the associated microbiota of *Mytilus* hybrids

#### Overall diversity of the cultivable microbial community

**Figure 2:**
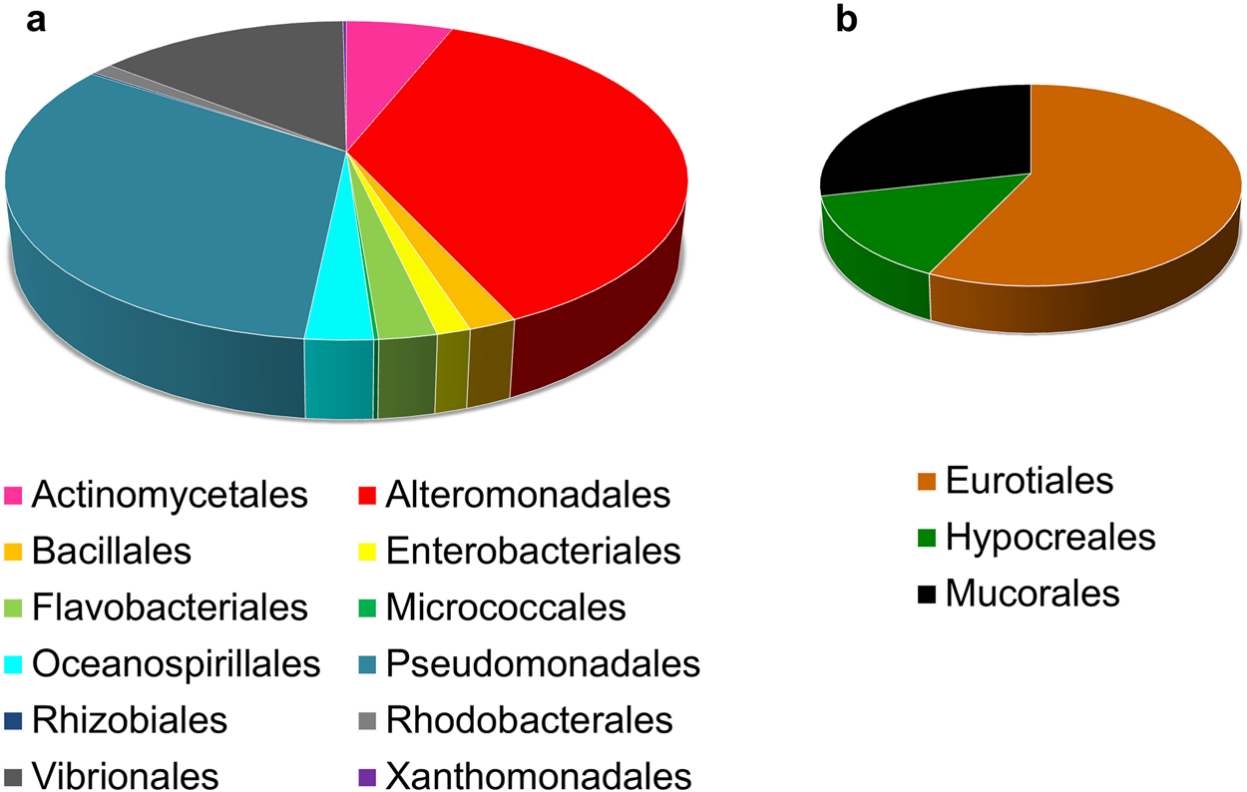
Proportions of microbial orders associated to *Mytilus* species. Microbial strain abundances are based on the sum of all strains affiliated to each microbial order based on 16S rRNA gene and ITS1–5.8S rRNA gene–ITS2 sequences, respectively. (**a**) Bacterial orders; (**b**) fungal orders.

The cultivation of associated microorganisms of each individual *Mytilus* spp. in five different agar media yielded in total 121 bacterial (94%) and fungal (6%) strains (see Supplementary Table S2). Microorganism distribution was calculated based on the taxonomic rank order (Fig. 2). In total, 15 microbial orders were obtained, representing 12 bacterial and three fungal orders. The two gammaproteobacterial orders Alteromonadales and Pseudomonadales were present in all tested specimens and dominated the cultivable microbiota in most of the mussels. Vibrionales (Gammaproteobacteria) and Actinomycetales (Actinobacteria) constituted 19.4% of the cultivable microbiota, thus representing the second-most abundant microbial orders. The mussel associated cultivable microbial diversity showed individual variability. In eight individuals (individuals 2, 5-10, 13) no Actinomycetales were found. Cultivation from individuals 7 and 9 resulted in representatives of only three different microbial orders, whereas eight were obtained from individuals 1 and 19 (see Supplementary Table S3).

**Figure 3:**
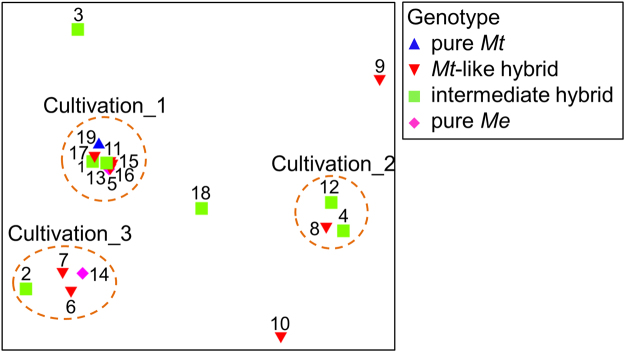
Analysis of the cultivable microbial community of *Mytilus* spp. individuals. 2D MDS plots were calculated on the basis of abundance data (CFU) of isolated bacterial and fungal orders. Mussel individuals are indicated by sample numbers 1–19 and the respective genetic background by differently colored shapes. Blue triangles: pure *Mt*; red triangles: *Mt*-like hybrid; green squares: intermediate hybrid; pink diamonds: pure *Me*. *Me*-like hybrid genotype is not represented because no individuals of that genotype were detected in this study (see Fig. 1). Three clusters are highlighted as “Cultivation_1”, “Cultivation_2” and “Cultivation_3”.

The observed inter-individual variation was corroborated by multidimensional scaling (Fig. 3). These data indicate that the mussels host a differentially structured microbial community, which could be assigned to one of three different “Cultivation” clusters (except for specimens 3, 9, 10 and 18). However, no genotype-specific association of microbes could be detected since mussel individuals within one cluster were assigned to either 2 (“Cultivation_2”), 3 (“Cultivation_3”) or all 4 (“Culivation_1”) different hybrid scores. Also, pure *Me* and *Mt*, reflecting the two most distant genotypes, could not be differentiated regarding their microbial association patterns since *i*.*e*. the *Me* individual 5 and *Mt* individual 19 were both in the same cluster (“Cultivation_1”).

#### Bacterial community fingerprinting by RFLP

RFLP analyses of the bacterial 16S rRNA gene resulted in a total of six differently sized fragments (see Supplementary Table S4). In most specimens, two different fragments could be detected, but no fragment was shared among all individuals. Only individual 6, 11 and 17 possessed three fragments/bands while individual 8 had four bands and one band D (700 bp) was solely present in individual 18.

**Figure 4:**
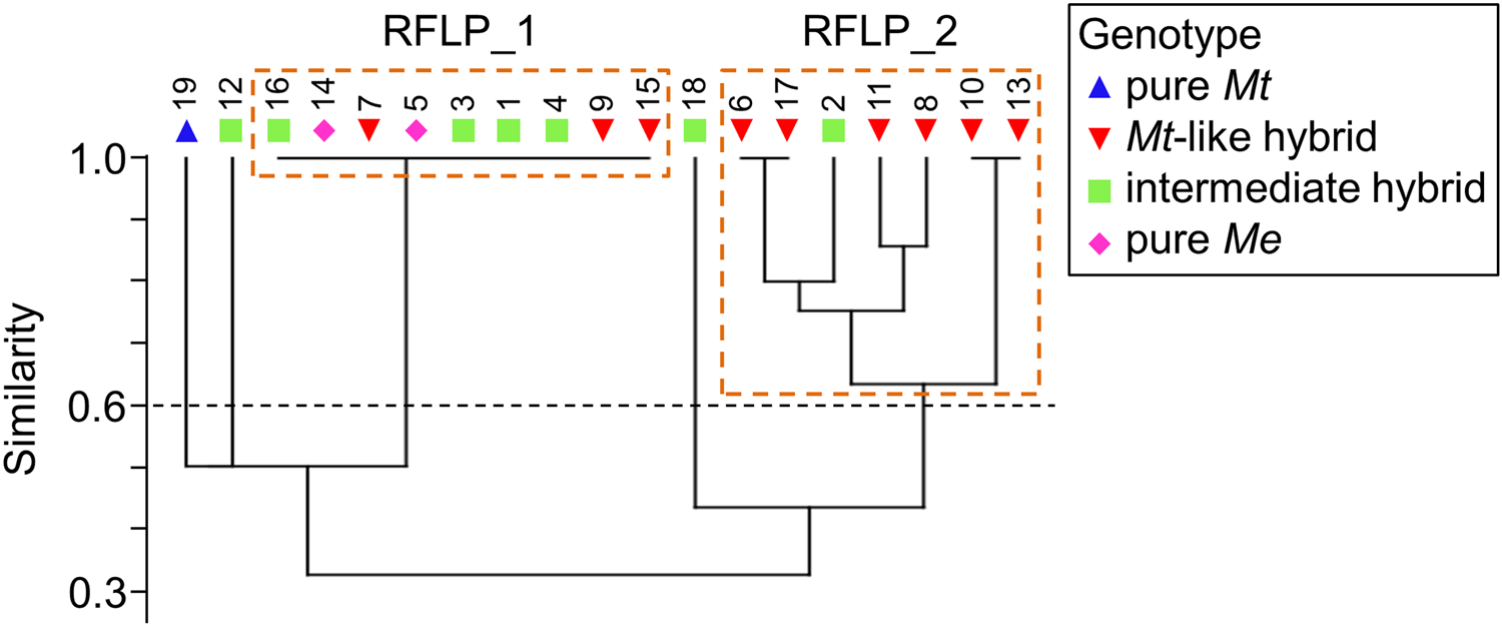
Cluster analysis of 16S rRNA gene-based RFLP community fingerprinting. Presence/absence patterns of bands were subjected to UPGMA cluster analysis. Mussel individuals are indicated by sample numbers 1–19 and the respective genetic background by differently colored shapes. Blue triangles: pure *Mt*; red triangles: *Mt*-like hybrid; green squares: intermediate hybrid; pink diamonds: pure *Me*. *Me*-like hybrid genotype is not represented because no individuals of that genotype were detected in this study (see Fig. 1). Two clusters with individuals of similar RFLP band patterns (threshold **>**60% similarity) are highlighted (“RFLP_1”, “RFLP_2”).

To compare relatedness between mussel individuals, RFLP fragment patterns were analysed by means of UPGMA clustering and mapped to the mussel genotype. The results were in accordance with findings from the cultivation-based study (Fig. 4). We found individual variability reflected in the presence of two different clusters (similarity >60%) but no genotype-specific associations. This finding is best exemplified by cluster “RFLP_1” that includes nine mussel individuals possessing identical RFLP band patterns but belonging to three different genotypes (pure *Me*, intermediate hybrid, *Mt*-like hybrid). Three specimens (individual 12, 18, 19) showed rather distant RFLP band patterns compared to all other individuals (similarity <50%), which was again not related to one specific genotype, but included pure *Mt* and intermediate hybrids.

### Metabolite profiling of KF blue mussels

The metabolite profiling of each individual mussel extract was carried out by UPLC-QTOF-MS/MS. Ethyl acetate (EtOAc) was used as extraction solvent, as preliminary optimisation studies revealed the highest chemical diversity. To ensure the optimal separation of closely eluting compounds, a moderately polar to very nonpolar solvent gradient was employed.

Most compounds eluted between 3.32 and 5.59 min and were consistently present in all mussel specimens. Qualitative global profiling of these extracts resulted in the detection of 76 different compounds, 65 of which were detected in all extracts (see Supplementary Table S5). We were able to annotate 40% of the compounds to putatively known natural products isolated from either *Mytilus* species, related marine animals, or from microorganisms (Table 2, see also Supplementary Table S5). Dereplication of the putatively known metabolites was based on their molecular weight, their polarity (retention time), their fragmentation pattern and the alignment of their calculated molecular formula to those available in the most comprehensive natural product databases (*e*.*g*. Dictionary of Natural Products (DNP) and Universal Natural Products Database (UNPD)). Referring to the minimum reporting standards for chemical analysis^45^, 28 metabolites were putatively annotated and assigned to identification confidence (IC) level 2. The remaining two metabolites (**16**, **45**) were characterised as level 3 compounds by determining the compound class. Table 2 shows the most common compounds detected and subsequently putatively annotated in all *Mytilus* extracts by UPLC-HRMS/MS. The putative identification of those major compounds was confirmed by comparing the experimentally determined fragmentation pattern (if detected) to the chemical structure of the known compound. Compound **22** was annotated as tetronolide (*m/z* [M + H]^+^ 553.2822, C_32_H_40_O_8_). The MS/MS fragmentation pattern showed three main fragments, two of them corresponding to the loss of CH_4_O (*m/z* [M + H]^+^ 521.2505) and C_22_H_28_O_8_ (*m/z* [M + H]^+^ 133.0859) consistent with the MS/MS predicted spectra obtained from Competitive Fragmentation Modeling for Metabolite Identification (CFM-ID). Chlorophyllone a (**43**, *m/z* [M + H]^+^ 533.2553, C_33_H_32_N_4_O_3_) was characterised by four main fragments corresponding to the loss of a molecule of H_2_O (*m/z* [M + H]^+^ 515.2399), either C_2_H_4_ or a carbonyl group (C=O) (m/z [M + H]^+^ 505.2548) and C_3_H_4_O_2_ (*m/z* [M + H]^+^ 461.2652) as well as the loss of CH_2_OH (*m/z* [M + H]^+^ 474.2371) from the ion fragment *m/z* 505.2548. The loss of either a C_2_H_4_ or a carbonyl group (C=O, *m/z* [M + H]^+^ 507.2710) from the parent mass of compound **44** (*m/z* [M + H]^+^ 535.2703, C_33_H_34_N_4_O_3_) led to the assignment to pyropheophorbide a. For compound **45** (*m/z* [M + H]^+^ 565.2457, C_40_H_52_O_2_) only one of the experimentally detected major fragments (*m/z* [M + H]^+^ 477.2602, corresponding to a loss of C_5_H_12_O) could be assigned to alloxanthin, hence compound **45** was annotated as alloxanthin analogue. The loss of a molecule of H_2_O (*m/z* [M + H]^+^ 503.2400), either C_2_H_4_ or a carbonyl group (C=O, *m/z* [M + H]^+^ 493.2548) and C_3_H_8_ (*m/z* [M + H]^+^ 477.2610) from the parent mass *m/z* [M + H]^+^ 521.2553 (C_32_H_32_N_4_O_3_) confirmed the putative annotation of compound **46** (a & b) to okaramine A or H. The applied collision energy resulted in one fragment for *m/z* [M + H]^+^ 621.3923 (compounds **48**, **49**) that could also be detected via the *in-silico* predicted fragmentation of 9-hydroxybafilomycin D (loss of C_4_H_12_O_2_, *m/z* [M + H]^+^ 529.3249 for **48**, 529.3254 for **49**).

**Table 2:** Main compounds (>20% relative intensity) putatively identified in all *Mytilus* spp. extracts using UHPLC-HRMS.

Peak number	*m/z* [M + H]^+^ (exp.)	m/z [M + H]^+^ (calc.)	Molecular formula	Δ ppm	Common (trivial) name	Chemical class
22	553.2822	553.2815	C_32_H_40_O_8_	0.5	Tetronolide	Macrolide
39	567.2967	567.2971	C_34_H_38_N_4_O_4_	1.1	Corallistin B	Porphyrin pigment
43	553.2552	533.2553	C_33_H_32_N_4_O_3_	0.2	Chlorophyllone a	Tetrapyrrole
44	535.2703	535.2709	C_33_H_34_N_4_O_3_	1.1	Pyropheophorbide a	Tetrapyrrole
45	565.2457	565.4046	C_40_H_52_O_2_	>5.0*	Alloxanthin analogue	Carotenoid
46a, 46b	521.2553	521.255	C_32_H_32_N_4_O_3_	0	Okaramine A or H	Indole diketopiperazine
48, 49	621.3923	621.3944	C_35_H_56_O_9_	>5.0*	9-Hydroxy-bafilomycin D	Macrolide

*m/z* [M + H]^ +^ (exp.): experimentally determined (obtained from base peak chromatograms), *m/z* [M + H]^ +^ (calc.): calculated from the assigned molecular formula, *peak in saturation (not possible to determine a ppm error below 5.0).

**Figure 5:**
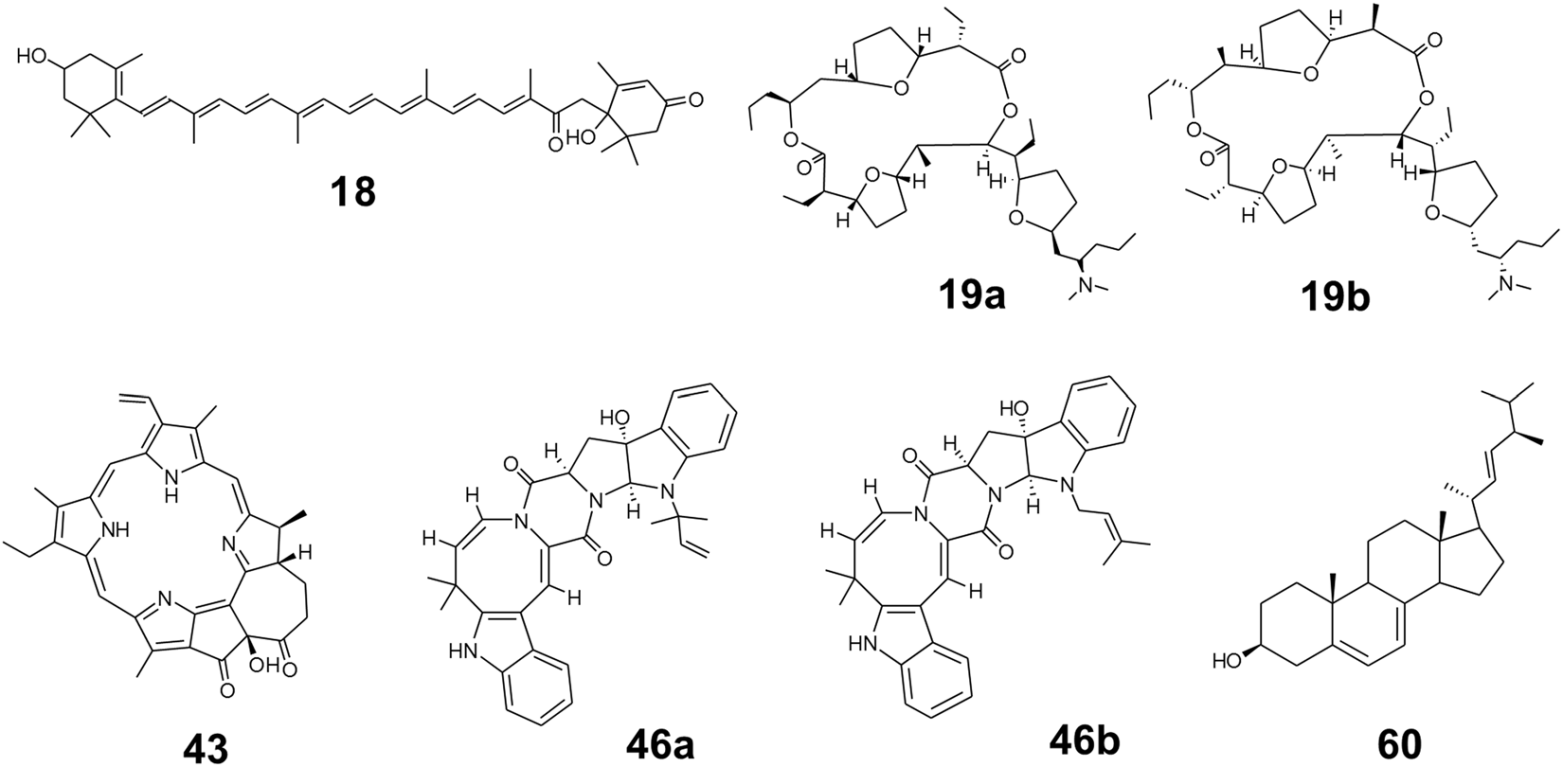
Diversity of chemical structures identified in extracts of blue mussels by UPLC-QTOF-MS/MS analysis. Exemplified structures are given with the respective peak number. Identity of all compounds identified in the extracts, characteristic MS data and related references can be found in the supplementary material (see Supplementary Table S5).

The chemical diversity of compounds found in the extracts was broad, with the most abundant chemical group being the pigments (13 putative hits), comprising mainly carotenoids and tetrapyrroles, also known as chlorophyll a derivatives (Fig. 5, see also Supplementary Table S5 and Supplementary Fig. S1). Eleven putatively annotated compounds belonged to the classes of alkaloids (**4**, **15**, **33**, **46a/b**) and polyketides (**19**, **22**, **31**, **48**, **49**, **54**, **70**). Two peptides (**13**, **64**), a polysaccharide (**38**) and a sterol (**60**) were putatively dereplicated (see Supplementary Table S5). Common free amino acids, sugars or large molecular weight antimicrobial peptides (AMPs, molecular weight >4 kDa) were not detected because of their hydrophilic nature, the application of EtOAc as solvent and the threshold set by the MS equipment (max. 1.6 kDa). The molecular formulae calculated for 46 compounds could not be assigned to any known natural product (IC level 4 compounds).

**Figure 6:**
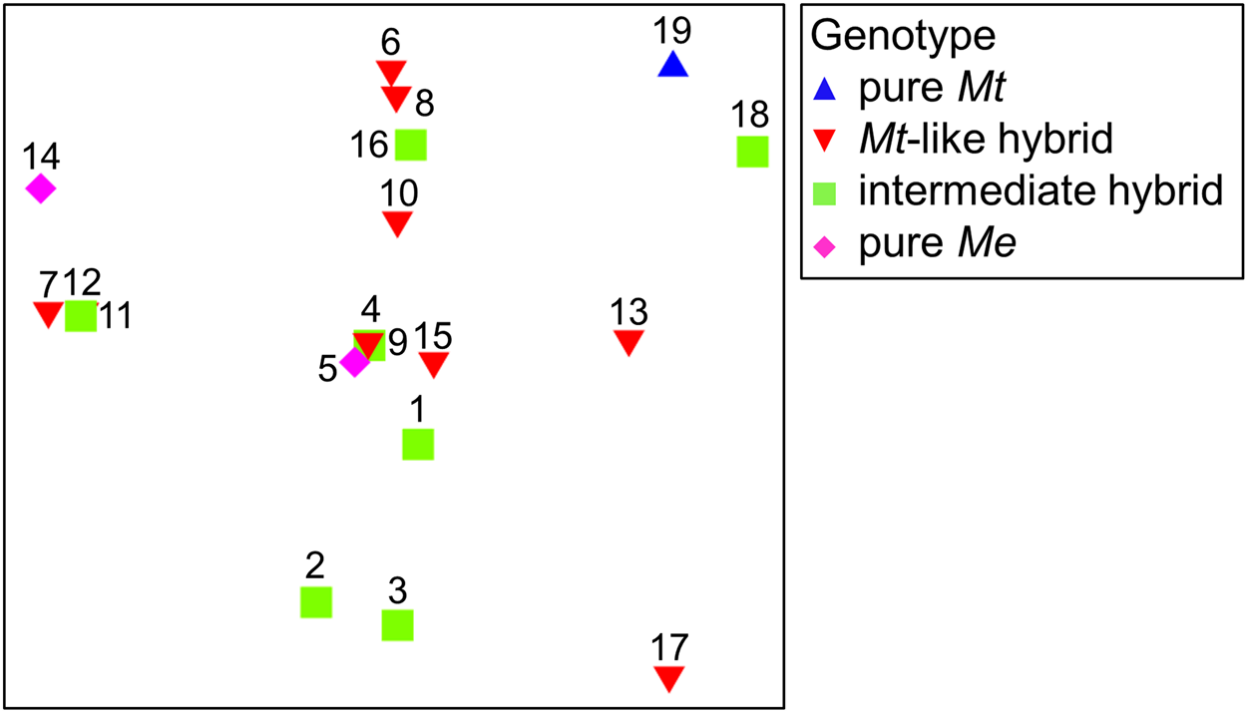
2D MDS of secondary metabolite profiles from farmed Baltic blue mussel extracts. The comparison of individual profiles is based on the presence/absence of each detected peak in UPLC-MS data. Mussel individuals are indicated by sample numbers 1–19 and the respective genetic background by differently colored shapes. Blue triangles: pure *Mt*, red triangles: *Mt*-like hybrid, green squares: intermediate hybrid, pink diamonds: pure *Me*. *Me*-like hybrid genotype is not represented because no individuals of that genotype were detected in this study (see Fig. 1).

We performed a comparison of metabolite profiles to the mussel individuals with respect to their genotype. Although we found some individual variation of metabolite profiles, a clear correlation between the metabolite profiles to genotypes was absent. For example, individuals 11 and 12, which belong to different genotypes, showed completely identical metabolite profiles (Fig. 6). Generally, regardless of the genotype, the metabolite profiles of mussel individuals clustered closely together without showing distinct clusters but the observed variation rather indicated inter-individual differences. For example, the metabolite profile from individual 5 (pure *Me*) clustered in close vicinity to specimens from both intermediate and *Mt*-like hybrids. Furthermore, the difference of the metabolite profiles of the pure *Me* individuals (5 & 14) is similar to the difference of both of them to the pure *Mt* individual (19).

## Discussion

Aquaculture KF mussel specimens were genotyped at the two nuclear loci EFbis and Glu-5′. Four different genotypes were detected, with the highest number of specimens being dominated by *Mt* alleles. Thus, our genotypic assessment confirmed the genetic complexity and dynamic nature of the Baltic Sea hybrid zone^16^, but contrasts with previous studies with respect to *Mt* allele frequencies in the KF^19^. Wildly growing *Mytilus* populations in KF were described to show a higher *Me* allele frequency when using the same markers and were classified as Baltic *Me*^19^ (Table 1). Regarding the marker Glu-5′, 80% of the genotyped individuals showed homozygosity for *Me* alleles^19^, which is far more compared to the detected allele frequencies in this study (47%). Only for the locus EFbis, a dominance for *Mt* alleles in KF population was also described, but with significantly lower frequencies of homozygous *Mt* alleles (between 50 and 62% in contrast to 84% in our study)^19^. The dominance of homozygous *Me* and strong introgression by *Me* alleles was shown for *Mytilus* populations found west of the Malmö-Stralsund transition zone using a number of different markers, such as SNPs^20^, ITS^16,46,47^ and MAL-1^16^ (see also Table 1). Based on their allelic state at EFbis and Glu-5′, this study confirms the hybrid character of KF *Mytilus* populations but provides first evidence that farmed blue mussels in KF showed an allele shift towards *Mt* alleles. These findings may serve as first evidence that the farmed KF blue mussel population is encountering a genetic shift towards a more *Mt*-like population. Phenotypic qualitative observations support this evidence. Mussels in the farm decreased in size and shells became more fragile during the last years. In accordance with our findings, two surveys showed that mussels’ fitness as described by wet weight, shell weight, flesh weight and shell stability was related to the genotype in farmed *Mytilus* species^7,44^. Mussels with fragile shells were shown to be *Mt*-like or intermediate hybrids^7^, whereas *Me* specimens grew faster and gained more weight^44^ at aquaculture sites in Scotland (UK) and Newfoundland (Canada). However, a correlation between the flesh (or dry weight) of the mussels to the respective genotype could not be detected in this study (Supplementary Fig. S2). Genotyping with the two nuclear markers yielded suitable insights into the genotypic distribution in the mussel farm and provided an adequate basis for comparing the microbiota and metabolome data sets. Future studies, including other marker systems such as SNP markers^10,11,14^, are needed to prove whether the observed predominance of *Mt* in farmed mussels can also be observed by using other genotyping markers or if the natural blue mussel populations in the KF have shifted.

This study describes for the first time the microbial community associated with farmed KF blue mussels. A broad diversity of bacteria and fungi was isolated from mussel flesh homogenate and assigned to 15 different microbial orders. The observed predominance of Gammaproteobacteria was in agreement with previous studies on blue mussels from other regions. Actinomycetales, Alteromonadales, Bacillales, Enterobacteriales, Flavobacteriales, Pseudomonadales and Vibrionales have also been obtained from either *Mt*^30^ or *Mg*^31,48,49^. Some representatives from Alpha- (orders Rhizobiales, Rhodobacterales) and Gammaproteobacteria (orders Oceanospirillales, Xanthomonadales) are being reported herein for the first time as part of the microbial community of *Mytilus* species. The seven fungal isolates, which belong to three different orders, accounted for only 6% of all detected microbes. The abundance of the fungal genera *Penicillium* (Eurotiales) and *Umbelopsis* (Mucorales) is in accordance with previous studies that investigated the associated fungal community of Algerian and French *Mytilus* species from aquaculture sites^28,50^. The present study provides first evidence for *Fusarium* sp. (order Hypocreales) as part of the microbial community of bivalves. Differences to other studies with respect to the occurrence of species may be due to the use of different isolation media as the medium composition is known to be a key selective factor in any cultivation study. In line with the published literature^28,30,31,48–50^, our study provides further evidence that farmed *Mytilus* spp. host several cultivable bacterial and fungal orders, which can be detected independently of the mussel cultivation site. In accordance with the metaorganism concept^22^, it can be assumed that some of the isolated microorganisms support the blue mussel’s health *e*.*g*. by exhibiting antibacterial defense. This has already been shown for a haemolymph-associated *Pseudoalteromonas* sp. strain obtained from French *Me* that possesses antibacterial activity against several aquaculture pathogens of the genera *Aeromonas* and *Vibrio*^29^.

The culture-independent examination of the microbial community associated with KF blue mussels was carried out through a RFLP analysis. Since the aim of this study was to provide mussel farms – in case of genotype specific patterns - with possible genotyping tools based on microbiota or metabolome features, RFLP analysis was selected as a well-established and easy to perform tool. Nonetheless, multivariate analysis of cultivation-dependent and -independent microbial profiling consistently showed no genotype-dependent variability of the *Mytilus*-associated microbiota and neither species- nor hybrid-specific microbial associations were detected (Figs 3 and 4). Stable species-specific microbial communities (core microbiome hypothesis) are common in marine and terrestrial animals^32,51^, as is the inter-individual variability^34,35^. The latter may exceed other dependencies of the microbiome, such as genotype or environmental influences^36^. In the case of Baltic blue mussels, the intensive hybridisation, which renders species-specific morphological characteristics, might be also rendering species-specific associations of microorganisms; thus only individual variability of the associated microbiota could be detected. It remains to be shown if the microbial community is dominated by intraspecific variations in other *Mytilus* spp. and hybrids. The current study demonstrated the importance of elucidating inter-individual variations when studying *e*.*g*. the effect of environmental factors or genetic traits on the microbial community^52,53^.

Research on *Mytilus*’ full metabolome is scarce and mainly focused on polar primary metabolites^5,37,43,54–57^. There is only one study on metabolic species discrimination that compared metabolite profiles of *Mg* and *Perna canaliculus* obtained by NMR (amino acids, sugars) and GC-MS (lipids)^43^. Our study is the first providing a detailed global metabolite analysis of *Mytilus* spp., in this case of farmed blue mussels from the Baltic Sea. Chemical profiling by UPLC-QTOF-MS/MS of individual whole tissue extracts revealed the presence of 76 compounds, almost half of which were putatively annotated at IC level 2 or 3 primary and secondary metabolites (see Supplementary Table S5)^45^. The extraction method, a single solvent and short chromatography time (in total 15 min), allowed identification of a broad diversity of known natural products (Fig. 5, see also Supplementary Fig. S1). LC-MS analysis showed that almost each individual possessed a unique metabolic profile (except for individuals 11 & 12). Eleven compounds, comprising various chemical groups (carotenoids, alkaloids, tyrosine derivatives) but also six non-identified compounds, were detected only in a few specimens (see Supplementary Table S5). The metabolite profiles could also not be related to the mussel genotypes. Few studies on *Mytilus* spp. detected metabolic differences at intraspecies level, *e*.*g*. caused by different gender^43,56^. In the present study, the mussel individuals were chosen randomly irrespective of their sex, which may influence their metabolic profile. Environmental influences such as physical properties of the water, diet, stress and disease can also modulate metabolite production^58^. Although the sampling procedure of this study was designed to avoid stress induced metabolite variations as much as possible, it might be another reason for variation^39,55^. Furthermore, the individually associated microbiota may influence the metabolome as already reported for tunicates and sponges^25,26^. The presence of individually different microbiota inducing differential reactions of the host may lead to individual mussel metabotypes^58^ *e*.*g*. production of defense metabolites against pathogenic microbiota in one individual versus production of metabolites enhancing settlement of beneficial microbiota in another. This is best exemplified by secondary metabolites that were putatively dereplicated herein, *e*.*g*. aspochalamin C (**4**), pamamycin 649 (**19a**, **19b**) and halstoctacosanolide C (**70**)^59–61^. These microorganism-derived metabolites are known for their activity against gram-positive bacteria and have been described from *Mytilus* species^28,50^. Comparisons to other *Mytilus* spp. such as *Mg* and *M*. *californianus* would be necessary to finally clarify genotypic influence on metabolite production. Similar to the results of the microbiota analysis, a marker compound for discrimination of different KF *Mytilus* spp. genotypes could not be identified. At this point, it should also be noted that the number of analysed individuals per genotype (1–9) was comparable to other studies that detected genotypic dependence of the associated microbiota^32,62,63^ or metabolome (3–12 per species/sampling location)^43,64,65^.

Eleven of the 76 identified compounds, such as the pigments alloxanthin (**50**, analogues: **16**, **46**), fucoxanthinol (**55**), amarouciaxanthin B (**18**) and the sterol ergosterol (**60**), were previously isolated from *Mytilus* species^66^. The largest proportion of the identified natural products has a microbial origin. Alkaloids and polyketides are not frequently reported from *Mytilus* spp. but in this study we detected eleven alkaloids and polyketides, which are known from sponges and marine derived microorganisms, indicating a microbial origin for most of the compounds. The alkaloids okaramine A (**46**) and renieramycin P (**33**) were originally reported from the fungi *Aspergillus* and *Penicillium* sp.^67,68^, while the polyketides pamamycin 649 A/B (**19**), tetronolide (**22**), antibiotic C 104 (**60**) and halstoctacosanolide C (**70**) originate from *Streptomyces* type Actinobacteria^60,61,69,70^. Likewise, two putatively annotated peptides acremolide D (**13**) and procidin S 735 (**64**) were reported from the *Mytilus* associated *Streptomyces* or *Acremonium* species^71,72^. The putative annotation of fungal metabolites originating from *Penicillium* sp. and other fungal species (not detected in this study) was expected, since they are well known from marine habitats or are described from *Mytilus* species^28,50,73,74^. The lack of these species in our study probably stemmed from the media used, since this culture-dependent study targeted largely bacteria and we used only one fungi-specific medium (modified Wickerham medium). Previous studies^28,50^ that used specific fungal growth media isolated over 100 fungal strains from *Me* and *Mg* indicating a high abundance and diversity of fungi in blue mussels. Additionally, the detection of fungal metabolites by the highly sensitive method UPLC-QTOF-MS/MS underlines the hypothesis of a higher prevalence of fungi. Overall 46 compounds could not be putatively assigned to any natural product already isolated from marine species or microbes. According to the minimum reporting standards recently implemented for metabolomics^45,75,76^, these compounds appear to be unknown (IC level 4) indicating the potential for biodiscovery of new natural products in *Mytilus* and its associated microbes.

## Conclusion

Farmed *Mytilus* spp. specimens from the Baltic Sea were genotyped as *Mt*-like population contrasting earlier results from wild KF populations. Microbial profiling of farmed *Mytilus* spp. revealed a rich associated microbial fauna that varied at individual level regardless of the mussel’s genotype. Most of the detected microorganisms have already been reported from *Mytilus* spp. in other regions but some orders such as Rhizobiales and Xanthomonadales were isolated for the first time from *Mytilus* species. We also compared individual metabolic profiles of the blue mussels obtained by UPLC-QTOF-MS/MS and concluded again genotype-independent variations. This study shows a large variety of natural products (*e*.*g*. pigments, alkaloids, polyketides, sterols) in Baltic *Mytilus* spp. extracts. The main driving factors of the observed individual differences in the microbiota and metabolome of the blue mussels remain to be unraveled. Pheno- and genotypic changes of *Mytilus* spp. due to aquaculture is of interest for mussel farmers, as product quality might be affected. Future studies should focus on product relevant parameters such as blue mussel flesh production and shell stability.

## Material and Methods

### Sampling and sample preparation

19 *Mytilus* spp. specimens were sampled individually in September 2015 at *Kieler Meeresfarm* GmbH (position: 54°22′30″N 10°09′47″E), an organic blue mussel farm located in KF (Baltic Sea, Germany; random sampling from different long lines). The mussels were cooled on ice during transport to the laboratory. Dissection of the mussel specimens was performed under sterile conditions. The whole flesh was removed from the shell and weighed. The tissue was placed into a sterile 15 mL reaction tube containing approximately 3.3 g of glass beads (0.5–2 mm diameter). The tubes were filled to a total of 14 mL with sterilised ultrapure water and shaken for 5 min at 1940 rpm (Reax Top, Heidolph Instruments GmbH & Co. KG, Schwabach, Germany). For molecular analysis, an aliquot of 500 µL was stored at −100 °C. For cultivation, another aliquot of 100 µL was diluted 1:10 with sterile ultrapure water. All remaining aliquots were stored at −100 °C (Ultra-Low Temperature Freezer MDF-1155, SANYO Electric Biomedical Co., Ltd., Japan).

### Genotyping

DNA extraction from the mussel individuals was performed using the InnuPREP DNA Mini Kit (Analytik Jena AG, Jena, Germany) according to manufacturer’s instructions. Mussel homogenate (100 µL) was thawed and immediately subjected to the lysis step. The lysis was performed overnight in lysis solution with Proteinase K at 50 °C and 600 rpm using a TMix 220 V thermomixer (Analytik Jena AG, Jena, Germany). The final step of the protocol (addition of 30 µL elution buffer) was performed twice in order to increase nucleic acid concentration. DNA of all samples was amplified by the use of two commonly used nuclear markers (modified after Stuckas *et al*.^19^: EFbis-forward 5′-fam-ACAAGATGGACAATACCGAACCACC-3′, EFbis-reverse 5′-CCT TCT GGA TTT CCA TGA ATC GG-3′^77^; Glu-5′-forward 5′-hex-CCAGTATACAAACCTGTGAAGA-3′, Glu-5′-reverse 5′-TGTTGTCTTAATAGGTTTGTAAGA-3′^78^) in a multiplex PCR reaction of 10 µL total volume, including primers (5 pmol), 5 µL QIAGEN Multiplex PCR Kit (QIAGEN, Hilden, Germany), 1 µL of 1:10 diluted DNA and 2 µL of DNA-free water. Amplification was performed in a T-1 Thermoblock (Biometra GmbH, Göttingen, Germany) and started with 15 min of initial denaturation at 95 °C. Initial denaturation was followed by 37 cycles of 94 °C for 30 sec, 57 °C for 90 sec and 72 °C for 1 min and was finalised by a final elongation step of 30 min at 60 °C. PCR products were analysed by automated capillary gel electrophoresis. For each sample, 8.75 µL Hi-Di formamide (Thermo Scientific, Schwerte, Germany) and 0.25 µL GeneScan 500 LIZ dye size standard (Thermo Scientific, Schwerte, Germany) were added to 1 µL PCR product into a 96-well plate. After denaturation at 95 °C for 2 min, the plate was sequenced by using a 3130*xl* Genetic Analyzer (Applied Biosystems Inc, California, USA). The results from the automated capillary gel electrophoresis were analysed with GeneMarker AFLP/Genotyping Software V1.91 (BioGene Ltd., Kimbolton, UK). DNA obtained from KF blue mussel specimens was analysed in comparison to DNA from the three pure species of the *Me* complex, *Me* (North Sea, Germany), *Mg* (Adriatic Sea, Croatia) and *Mt* (North Pacific, Washington, USA) (provided by Senckenberg Natural History Collection of Dresden, Germany). Based on the reference samples, an allele panel was compiled including all alleles and their size ranges detected for *Me*, *Mg* and *Mt*. The constructed allele panel served as the basis for genetic analysis of the 19 mussel individuals. A hybrid score was calculated for each specimen by counting alleles referred to as *Me*. Thus, hybrid scores ranged from 0 (pure *Mt*) to 4 (pure *Me*), whereas a score of 2 is hereafter described as intermediate hybrid. As no alleles specific for *Mg* were detected in any of the 19 individuals, *Mg* was not considered for the calculation of the hybrid score.

### Culture-dependent study of the microbial community

Ten-fold diluted mussel flesh homogenate of each individual was plated onto the following five different agar media (1.5% Bacto agar each): Hastings medium (0.93% Na_2_HPO_4_ × 12 H_2_O, 0.1% KH_2_PO_4_, 0.06% (NH_4_)H_2_PO_4_, 0.01% MgSO_4_, 2% NaCl, 0.5% tryptone, 0.3% Bacto yeast extract, 0.2% glycerol, pH 7.6), MacConkey-Agar (Carl Roth, Karlsruhe, Germany), *Mytilus*-medium (3% mussel homogenate from *Kieler Meeresfarm* GmbH, 1% sea salt (Instant ocean, Virginia, USA)), TSB12 + 3 medium (1.2% Bacto tryptic soy broth, 0.3% sodium chloride) and modified Wickerham medium^79^. The inoculated petri dishes were incubated in dark for nine days at 22 °C. All plates were checked for the colony forming units (CFU) of different morphotypes. Different morphotypes were transferred to new medium until pure cultures were obtained. Isolates were classified as bacteria or fungi by means of macro- and microscopic observation and by molecular identification. Therefore, DNA of all bacterial isolates was extracted by a freeze and thaw procedure. Cell material was put into 100 µL of DNA-free water, frozen overnight at −20 °C and finally thawed for 15 min at 99 °C. The suspension was centrifuged at 4 °C for 10 min at 8917 rpm (Biofuge fresco, Thermo Scientific, Osterode, Germany). The supernatant was transferred into a sterile 200 µL reaction tube and samples with DNA concentrations above 300 ng/µL were diluted to approximately 100 ng/µL. Fungal DNA was extracted using innuSPEED Lysis Tubes S (Analytik Jena, Jena, Germany). For that purpose, 400 µL of DNA-free water and one piece of overgrown agar were placed into a lysis tube. The tubes were shaken for 6 min at a frequency of 30/sec in a mixer mill MM 200 (Retsch, Hahn, Germany) and the DNA-containing supernatant was gained by centrifugation for 5 min at 8917 rpm (Biofuge fresco) and room temperature. Bacterial DNA was amplified using Eub27f and either 1492r or 1387r primers for amplification of the 16S rRNA gene^80^. Fungal DNA was amplified using ITS1 and ITS4 primers targeting the internal transcribed spacer (ITS) 1 and 2 interspersed by the 5.8S rRNA gene^81,82^. DreamTaq Green PCR Master Mix (2×) (Thermo Scientific, Schwerte, Germany) was mixed with approximately 100 ng DNA and 10 pmol of each primer. PCR for amplification of bacterial DNA was done in 30 cycles of each 30 sec of 92 °C denaturation, 55 °C annealing and 72 °C elongation. Final elongation was conducted for 5 min at 72 °C. PCR for amplification of fungal DNA was modified after Gardes and Bruns^83^ as follows: the second set comprised 11 repetition steps and the third set comprised 8 repetition steps. PCR products were analysed by Sanger sequencing^84^ at IKMB (Institute of Clinical Molecular Biology, Kiel University, Germany), using the primer 1387r for bacterial strains and ITS1 for fungal strains. Sequences were processed by ChromasPro V1.33 (Technelysium Pty Ltd, South Brisbane, Australia) and submitted to BLAST^85^ (Basic Local Alignment Search Tool) at NCBI (National Center for Biotechnology Information). DNA sequences of all microbial isolates are available in GenBank under the accession numbers MH109373-MH109379 (SUB3838348; fungi), MH109387-MH109499 and MH124166 (SUB3832608, SUB3843849; bacteria).

### Microbial community fingerprinting by 16S rRNA based RFLP

DNA extracted for the genotypic assessment of blue mussels was purified with the innuPREP DNA Mini Kit (Analytik Jena, Jena, Germany), starting with the addition of binding solution TBS and adjusted to the elution volume. Thus, 50 µL TBS, 100 µL HS and 150 µL MS were inserted following the manufacturer’s instructions. For restriction fragment length polymorphism (RFLP) technique, DNA was amplified using the following re-amplification protocol. PCR was performed in a total volume of 20 µL, including eubacterial primers (Eub27f and 1492r, each 10 pmol), 10 µL innuMIX Green PCR Master Mix (Analytik Jena, Jena, Germany), DNA-free water and 1 µL of DNA. 8 µL of the PCR product were used as template for the re-amplification using PCR conditions previously described^86^. PCR products were dried at 40 °C for one hour in a rotational vacuum concentrator (RVC 2-33 CDplus, Martin Christ Gefriertrocknungsanlagen, Osterode, Germany) and dissolved in 17.3 µL of DNA-free water. Restriction digestion was performed using 0.2 µL BSA (bovine serum albumin), 0.5 µL *Msp*I (Promega, Hildesheim, Germany) and 2 µL 10× Buffer B (R002A). Restriction was performed at 37 °C for four hours and analysed by agarose gel electrophoresis (100 V, 1 h run time) applying 12 µL from the restriction digestion to a 1.25% gel containing SYBR Safe DNA Gel Stain 10.000× (Invitrogen, Life Technologies, Darmstadt, Germany). For band calling, a grid was placed over each gel image in order to evaluate the band patterns (only fragments >500 bp were taken into account) resulting in a presence (assigned “1”) /absence (assigned “0”) matrix.

### Extraction and metabolite profiling

Individual blue mussel homogenates were freeze-dried for two days (average yield 16.1% from dissected flesh). To remove sea salts, the freeze-dried material was homogenised in ultrapure water (8 mL) by a T 25 basic Ultra-Turrax (IKA-Werke, Staufen, Germany) at 17.500 rpm and the supernatant was discarded after centrifugation at 4700 rpm (Multifuge 1 L-R, rotor 75002002 G, Thermo Scientific, Osterode, Germany) for 10 min. The pellet was re-suspended in 6 mL EtOAc and washed again with 3 mL ultrapure water followed by a second centrifugation step (4700 rpm, 5 min). The EtOAc phase was evaporated to dryness at 40 °C using a rotary evaporator. For UPLC-MS/MS analyses, each EtOAc extract was dissolved in methanol (final concentration 1.0 mg/mL) and filtered through a 0.2 µm PTFE syringe filter (Carl Roth, Karlsruhe, Germany).

All samples were analysed by Acquity UPLC I-Class System coupled to Xevo G2-XS QTof Mass Spectrometer (Waters®, Massachusetts, USA). The extracts were injected (1.0 µL) into an Acquity UPLC® HSS T3 column (High Strength Silica C18, 1.8 µm, 2.1 × 100 mm, Waters®, operating at 60 °C). A binary mobile phase system (A: 0.1% formic acid in milliQ-water, B: 0,1% formic acid in acetonitrile (ULC-MS grade from Biosolve Chimie, Dieuze, France)) was pumped at a flow rate of 0.6 mL/min applying the following gradient (% of A given): initial, 50%; 4 min, 14%; 9–11 min, 5%; followed by washing and reconditioning of the column. The total run time was 15 min. The MS and MS^n^ spectra, in positive mode, were recorded using the following conditions: capillary voltage: 0.8 kV, cone voltage: 40 V, source temperature: 150 °C, cone gas flow: 50 L/h, desolvation gas flow: 1000 L/h and a collision energy ramp with low collision energy: 6–60 eV and high collision energy: 9–80 eV. Scan times were 0.1 s and acquisition range was *m/z* 50–1600. MassLynx® Software (version 4.1) was used for data acquisition such as determining the elemental composition of the detected compounds. Additionally, MS spectra were subjected to the classical databases Dictionary of Natural Products (DNP, http://dnp.chemnetbase.com), Universal Natural Product Database (UNPD, http://pkuxxj.pku.edu.cn/UNPD/), Reaxys (https://www.reaxys.com) and ChemSpider (http://www.chemspider.com) in order to identify putative known compounds. As a second step, the fragmentation pattern of putatively annotated compounds was matched with the structure of the respective known compounds. Fragments were either annotated manually or by the web-based *in silico* prediction tool CFM-ID (http://cfmid.wishartlab.com/predict)^87^. After the manual dereplication based on database comparison and the assignment of detected fragmentation patterns to known compounds, putatively annotated compounds were characterised by an identification confidence level ranging in this study from 2 to 4^45,75^. Level 2 and 3 compounds are putatively annotated compounds or compound classes, level 4 compounds are unknown compounds because their molecular weight, putative molecular formula and detected fragmentation pattern do not match with any known natural product or compound class.

Compound lists for subsequent multivariate analysis were compiled for each mussel individual separately. These lists comprised the following data for each detected peak: the retention time (RT in min), the *m/z* [M + H]^+^ value (experimentally determined, obtained from base peak chromatograms), the calculated molecular formula and the *m/z* [M + H]^+^ value calculated from the latter. To avoid detection of peaks that resulted from injection or column bleeding, all compounds detected before 1 min or after 10 min and 30 sec were excluded from the analysis.

### Statistics

All statistics were performed with Microsoft Office Excel 2010 and Past 3.12^88^ (https://folk.uio.no/ohammer/past/). For microbial community analysis, data of the taxonomic rank order were used for statistical comparison. Abundance matrices were constructed and square root transformed (see Supplementary Table S3). Comparison was achieved by computing 2D non-metric MDS plots based on the Bray-Curtis similarity index. Presence/absence matrices obtained by the RFLP (see Supplementary Table S4) were statistically compared using the hierarchical UPGMA (Unweighted Pair Group Method with Arithmetic mean) clustering method, based on the Bray-Curtis similarity index. Metabolite profiles (compound lists) were compared among the mussel specimens by compiling 2D non-metric MDS plots (Bray-Curtis similarity index). The dataset used for this statistical comparison was a presence/absence matrix of the detected metabolites based on RT and *m/z* value.

### Data availability statement

The datasets generated and analysed during the current study are available from the corresponding author on reasonable request.

## Electronic supplementary material

Supplementary information
