# Supplementary material for: Combined genotyping, microbial diversity and metabolite profiling studies on farmed Mytilus spp. from Kiel Fjord
Source: Sci Rep. 2018 May 22;8:7983. doi: 10.1038/s41598-018-26177-y (PMC5964093; doi:10.1038/s41598-018-26177-y)
Supplement: Supplementary file 1 — Supplementary information [file 41598_2018_26177_MOESM1_ESM.pdf]

Supplementary information

Combined genotyping, microbial diversity and metabolite profiling studies on farmed *Mytilus* spp. from Kiel Fjord

Caroline Utermann[1], Delphine Parrot[1], Corinna Breusing[2,5], Heiko Stuckas[3], Tim Staufenberger[4], Martina Blümel[1], Antje Labes[1,6], Deniz Tasdemir[1*]

[1]GEOMAR Centre for Marine Biotechnology (GEOMAR-Biotech), Research Unit Marine Natural Products Chemistry, GEOMAR Helmholtz Centre for Ocean Research Kiel, Am Kiel-Kanal 44, 24106 Kiel, Germany

[2]Research Unit Evolutionary Ecology of Marine Fishes, GEOMAR Helmholtz Centre for Ocean Research Kiel, Duesternbrooker Weg 20, 24105 Kiel, Germany

[3]Senckenberg Natural History Collection Dresden, Population Genetics, Koenigsbruecker Landstrasse 159, 01109 Dresden, Germany

[4]Kieler Meeresfarm GmbH, Richthofenstrasse 31, 24159 Kiel, Germany

[5]Present Address: Monterey Bay Aquarium Research Institute, 7700 Sandholdt Road, Moss Landing, California 95039, USA

[6]Present Address: Flensburg University of Applied Sciences, Kanzleistrasse 91–93, 24943 Flensburg, Germany

[*] Corresponding author: Deniz Tasdemir

Email: dtasdemir@geomar.de

**Supplementary information**	**Page**
Table S1. Genotypic assessment of farmed *Mytilus* spp. individuals with two nuclear markers.	3
Table S2. Origin and identification of all microbial strains isolated from *Mytilus* species.	4-8
Table S3. Distribution of CFU among the 19 *Mytilus* spp. individuals.	9
Table S4. Analysis of RFLP band patterns of 19 *Mytilus* spp. individuals.	10
Table S5. Putative identification of metabolites detected in farmed KF *Mytilus* species.	11-15
Figure S1. Chemical structures of identified compounds in extracts of blue mussels by UPLC-QTOF-MS/MS analysis.	16-18
Figure S2. Figure 1. Distribution of fresh and dry weight among the different genotypes (n=18).	19
Additional references for supplementary information (accompany Table S5).	20-21

Table S1. Genotypic assessment of farmed *Mytilus* spp. individuals with two nuclear markers. *Me*: *M. edulis, Mt*: *M. trossulus*, E: *Me* allele, T: *Mt* allele. The sizes of the amplified marker genes were the following: EFbis *Me*: 356 bp, EFbis *Mt*: 300 bp, Glu-5' *Me*: 177.5 bp, Glu-5' *Mt*: 165.5 bp.

**Individual**	**EFbis**		**Glu-5'**		**Hybrid score**	**Genotypic determination**
1	T	T	E	E	2	Intermediate hybrid
2	T	T	E	E	2	Intermediate hybrid
3	T	T	E	E	2	Intermediate hybrid
4	T	T	E	E	2	Intermediate hybrid
5	E	E	E	E	4	Pure *Me*
6	T	T	E	T	1	*Mt*-like hybrid
7	T	T	E	T	1	*Mt*-like hybrid
8	T	T	E	T	1	*Mt*-like hybrid
9	T	T	E	T	1	*Mt*-like hybrid
10	T	T	E	T	1	*Mt*-like hybrid
11	T	T	E	T	1	*Mt*-like hybrid
12	T	T	E	E	2	Intermediate hybrid
13	T	T	E	T	1	*Mt*-like hybrid
14	E	E	E	E	4	Pure *Me*
15	T	T	E	T	1	*Mt*-like hybrid
16	T	T	E	E	2	Intermediate hybrid
17	T	T	E	T	1	*Mt*-like hybrid
18	T	T	E	E	2	Intermediate hybrid
19	T	T	T	T	0	Pure *Mt*

Table S2. Origin and identification of all microbial strains isolated from *Mytilus* species. Microbial strains are listed by their strain number, accession number and given with their identity (next related BLAST hits). For strains with >4 possible genera only the most likely microbial genus is presented. It is indicated from which mussel individual and medium each isolate was obtained. H: Hastings medium, M: *Mytilus*-medium, MC: MacConkey-Agar, T: TSB3+12, W: modified Wickerham medium.

Strain no.	Accession no.	Identity	Microbial order	Mussel individual(s)	Medium
MT2	MH109391	*Pseudoalteromonas* sp.	Alteromonadales	1, 13,14	H
MT3	MH109392	*Pseudoalteromonas* sp.	Alteromonadales	1, 2, 3, 5, 10, 17	H
MT4	MH109393	*Vibrio aestuarianus*	Vibrionales	1, 8, 17	H
MT6	MH109394	*Pseudoalteromonas* sp.	Alteromonadales	1, 3, 5, 9-11, 13, 14	H
MT7	MH109395	*Bacillus* sp.	Bacillales	1	H
MT8	MH109396	*Pseudomonas* sp.	Pseudomonadales	1, 2, 4-6, 8, 10, 12-19	T
MT10	MH109397	*Pseudoalteromonas* sp.	Alteromonadales	1-3, 5-7, 11, 13, 15, 17, 19	T
MT13	MH109398	*Pseudomonas* sp.	Pseudomonadales	1-6, 8, 11, 12, 14-18	MC
MT15	MH109399	*Exiguobacterium* sp.	Bacillales	2, 6	H
MT16	MH109400	*Erwinia* sp.	Enterobacteriales	2	H
MT17	MH109401	*Flavobacterium* sp.	Flavobacteriales	2, 4, 8, 10, 11, 13, 14, 19	T
MT18	MH124166	Vibrionaceae (*Grimontia marina, Photobacterium* sp., *Vibrio* sp.)	Vibrionales	2, 5	T
MT19	MH109402	*Vibrio* sp.	Vibrionales	2	T
MT20	MH109379	*Fusarium* sp.	Hypocreales	18	W
MT21	MH109403	*Vibrio hispanicus*	Vibrionales	2	M
MT22	MH109404	*Pseudoalteromonas* sp.	Alteromonadales	15	H
MT26	MH109405	*Shewanella* sp.	Alteromonadales	3	MC
MT28	MH109406	*Pseudoalteromonas* sp.	Alteromonadales	3, 15	H
MT29	MH109407	*Kocuria rhizophila*	Actinomycetales	1, 3, 4, 11, 15, 16, 18, 19	H
MT30	MH109408	*Pseudoalteromonas* sp.	Alteromonadales	3	H
MT31	MH109409	Halomonadaceae (*Cobetia* sp., *Halomonas* sp.)	Oceanospirillales	3	W
MT33	MH109410	*Pseudoalteromonas* sp.	Alteromonadales	3, 8, 9, 15, 18 , 19	M
MT34	MH109411	Enterobacteriaceae (*Cedecea* sp., *Citrobacter* sp., *Enterobacter* sp., *Leclercia adecarboxylata*	Enterobacteriales	4, 5	MC
MT38	MH109412	*Pseudoalteromonas* sp.	Alteromonadales	5	H

Strain no.	Accession no.	Identity	Microbial order	Mussel individual(s)	Medium
MT42	MH109413	*Vibrio hispanicus*	Vibrionales	6, 7, 14	M
MT44	MH109414	*Pseudomonas* sp.	Pseudomonadales	8	MC
MT46	MH109415	*Pseudoalteromonas* sp.	Alteromonadales	10	MC
MT47	MH109416	*Vibrio hispanicus*	Vibrionales	1, 4, 6, 10, 12, 14, 18	W
MT48	MH109417	*Flavobacterium jumunjinense*	Flavobacteriales	10	T
MT49	MH109418	*Shewanella* sp.	Alteromonadales	11, 14, 16, 17	M
MT50	MH109378	*Umbelopsis* sp.	Mucorales	4	W
MT53	MH109375	*Penicillium* sp.	Eurotiales	19	H
MT54	MH109419	*Shewanella* sp.	Alteromonadales	12	MC
MT55	MH109420	Halomonadaceae (*Cobetia* sp., *Halomonas* sp.)	Oceanospirillales	12	MC
MT56	MH109421	*Bacillus* sp.	Bacillales	6, 12	W
MT57	MH109376	*Penicillium* sp.	Eurotiales	12	H
MT58	MH109422	*Psychrobacter* sp.	Pseudomonadales	14	W
MT59	MH109423	Halomonadaceae (*Cobetia* sp., *Halomonas* sp.)	Oceanospirillales	11	W
MT61	MH109424	*Vibrio aestuarianus*	Vibrionales	14, 15, 18	T
MT62	MH109425	*Pseudoalteromonas* sp.	Alteromonadales	5	H
MT63	MH109426	*Shewanella* sp.	Alteromonadales	14	MC
MT65	MH109427	*Flavobacterium jumunjinense*	Flavobacteriales	14	T
MT66	MH109428	*Pseudoalteromonas tunicata*	Alteromonadales	14	T
MT67	MH109429	*Vibrio hispanicus*	Vibrionales	1-3, 5, 6, 8, 10, 13-15, 17-19	T
MT73	MH109430	*Pseudoalteromonas* sp.	Alteromonadales	16	H
MT75	MH109431	Vibrionaceae (*Aliivibrio* sp, *Vibrio* sp.	Vibrionales	16	H
MT79	MH109432	*Pseudoalteromonas* sp.	Alteromonadales	17, 18	H
MT80	MH109433	*Pseudoalteromonas* sp.	Alteromonadales	17	H
MT86	MH109434	*Acinetobacter* sp.	Pseudomonadales	18	MC
MT88	MH109435	*Pseudoalteromonas* sp.	Alteromonadales	18	H
MT90	MH109436	Flavobacteriaceae (*e.g. Bizonia* sp.)	Flavobacteriales	8, 11	H
MT95	MH109437	*Shewanella* sp.	Alteromonadales	19	T
MT101	MH109438	*Pseudoalteromonas* sp.	Alteromonadales	1	H
MT102	MH109439	*Psychrobacter* sp.	Pseudomonadales	2, 9, 19	H
MT105	MH109440	*Psychrobacter* sp.	Pseudomonadales	2, 19	H

Strain no.	Accession no.	Identity	Microbial order	Mussel individual(s)	Medium
MT106	MH109441	*Vibrio* sp.	Vibrionales	2	H
MT108	MH109442	*Pseudomonas* sp.	Pseudomonadales	12, 18	H
MT109	MH109443	*Pseudoalteromonas* sp.	Alteromonadales	18	H
MT110	MH109444	*Pseudomonas* sp.	Pseudomonadales	2-6, 9, 11, 13, 17	H
MT111	MH109445	*Pseudomonas* sp.	Pseudomonadales	5, 6, 15	H
MT112	MH109446	*Microbacterium* sp.	Actinomycetales	17	H
MT115	MH109447	*Pseudomonas* sp.	Pseudomonadales	6, 9, 10, 12, 17	H
MT120	MH109448	*Pseudoalteromonas* sp.	Alteromonadales	8	H
MT124	MH109449	*Pseudoalteromonas* sp.	Alteromonadales	13	H
MT126	MH109450	*Micrococcus* sp.	Actinomycetales	14	H
MT127	MH109451	*Pseudoalteromonas* sp.	Alteromonadales	15	H
MT128	MH109452	*Vibrio* sp.	Vibrionales	14, 16	H
MT129	MH109453	*Psychrobacter* sp.	Pseudomonadales	8, 19	H
MT130	MH109454	*Pseudomonas* sp.	Pseudomonadales	18	H
MT132	MH109455	Rhodobacteraceae (*Albirhodobacter* sp., *Paracoccus* sp., *Rhodobacter* sp., *Roseicitreum* sp.)	Rhodobacterales	19	H
MT133	MH109456	*Pseudomonas* sp.	Pseudomonadales	3-6, 8-15, 17, 18	T
MT136	MH109457	*Psychrobacter* sp.	Pseudomonadales	3, 4, 8, 9, 11-16, 18	T
MT137	MH109458	*Psychrobacter* sp.	Pseudomonadales	10, 19	T
MT141	MH109459	*Vibrio metschnikovii*	Vibrionales	4	T
MT143	MH109460	*Pseudomonas* sp.	Pseudomonadales	5, 9	T
MT145	MH109461	*Paracoccus yeei*	Rhodobacterales	9, 13	T
MT146	MH109462	*Pseudomonas* sp.	Pseudomonadales	10	T
MT149	MH109463	*Microbacterium* sp.	Actinomycetales	12	T
MT152	MH109377	*Penicillium* sp.	Eurotiales	17	T
MT156	MH109464	*Pseudomonas* sp.	Pseudomonadales	2-8, 11, 12, 14-17, 19	MC
MT157	MH109465	*Pseudomonas* sp.	Pseudomonadales	10	MC
MT160	MH109466	*Pseudomonas* sp.	Pseudomonadales	3, 5, 8, 12, 13, 15	MC
MT161	MH109467	*Shewanella* sp.	Alteromonadales	4, 14	MC
MT162	MH109468	*Pseudomonas* sp.	Pseudomonadales	4	MC
MT163	MH109469	*Serratia* sp.	Enterobacteriales	5	MC

Strain no.	Accession no.	Identity	Microbial order	Mussel individual(s)	Medium
MT165	MH109470	*Pseudomonas* sp.	Pseudomonadales	8	MC
MT166	MH109471	*Psychrobacter* sp.	Pseudomonadales	10, 18	MC
MT167	MH109472	*Shewanella* sp.	Alteromonadales	12, 19	MC
MT170	MH109473	*Pseudomonas* sp.	Pseudomonadales	12	MC
MT173	MH109474	*Pseudomonas* sp.	Pseudomonadales	12	MC
MT175	MH109475	*Pseudomonas* sp.	Pseudomonadales	15	MC
MT179	MH109476	*Arthrobacter psychrolactophilus*	Actinomycetales	1, 16	W
MT180	MH109477	*Pseudoalteromonas* sp.	Alteromonadales	1, 10, 11, 14	W
MT181	MH109478	Micrococcales (*e.g. Luteimicrobium* sp.)	Micrococcales	1	W
MT195	MH109479	*Paracoccus yeei*	Rhodobacterales	9	W
MT201	MH109480	*Microbacterium* sp.	Actinomycetales	14	W
MT202	MH109481	*Psychrobacter* sp.	Pseudomonadales	15	W
MT203	MH109482	*Aurantimonas* sp.	Rhizobiales	15	W
MT204	MH109483	Microbacteriaceae (*Agreia* sp., *Leifsonia* sp., *Salinibacterium* sp.,	Actinomycetales	15	W
MT205	MH109484	*Stenotrophomonas* sp.	Xanthomonadales	15	W
MT206	MH109485	*Streptomyces* sp.	Actinomycetales	16	W
MT209	MH109486	*Psychrobacter* sp.	Pseudomonadales	19	W
MT211	MH109487	*Pseudoalteromonas* sp.	Alteromonadales	1-4, 8, 11, 15, 19	M
MT212	MH109488	Enterobacteriaceae (*e.g. Enterobacter* sp.)	Enterobacteriales	1, 3, 4	M
MT213	MH109489	*Shewanella* sp.	Alteromonadales	1, 3, 17	M
MT214	MH109490	Halomonadaceae (*Cobetia* sp., *Halomonas* sp.)	Oceanospirillales	1, 3, 6, 13-15, 19	M
MT216	MH109491	*Bacillus* sp.	Bacillales	2	M
MT217	MH109492	*Shewanella* sp.	Alteromonadales	2, 3, 11, 13, 17, 19	M
MT219	MH109493	*Pseudomonas* sp.	Pseudomonadales	2, 3, 13, 15	M
MT221	MH109494	*Psychrobacter* sp.	Pseudomonadales	2	M
MT227	MH109495	*Pseudomonas* sp.	Pseudomonadales	3	M
MT228	MH109373	*Umbelopsis* sp.	Mucorales	4	M
MT232	MH109496	*Psychrobacter* sp.	Pseudomonadales	5, 15, 19	M
MT235	MH109497	*Pseudomonas* sp.	Pseudomonadales	9	M
MT237	MH109498	*Pseudomonas* sp.	Pseudomonadales	10, 15	M

Strain no.	Accession no.	Identity	Microbial order	Mussel individual(s)	Medium
MT238	MH109374	*Penicillium* sp.	Eurotiales	14	M
MT242	MH109499	*Dietzia* sp.	Actinomycetales	15	M
MT1A	MH109387	*Pseudoalteromonas* sp.	Alteromonadales	1, 5, 6, 8, 10, 12-19	H
MT4A	MH109388	*Vibrio* sp.	Vibrionales	2, 6, 7, 13, 14	H
MT4B	MH109389	*Pseudomonas* sp.	Pseudomonadales	1-4, 6, 8, 10-15, 18, 19	H
MT5C	MH109390	*Pseudoalteromonas* sp.	Alteromonadales	1, 3, 5, 12-15, 18	H

Table S3. Distribution of CFU among the 19 *Mytilus* spp. individuals. CFU of each individual microbial order are given for each individual separately and are displayed as square root transformed.

	Mussel individual																		
**Microbial order**	1	2	3	4	5	6	7	8	9	10	11	12	13	14	15	16	17	18	19
Bacteria																			
Actinomycetales	2.6	0.0	32.3	1.0	0.0	0.0	0.0	0.0	0.0	0.0	1.0	1.4	0.0	1.7	1.7	2.8	1.0	1.0	14.2
Alteromonadales	45.3	31.8	39.2	2.0	31.8	31.8	31.6	3.7	2.0	4.2	34.4	3.6	31.8	31.8	33.9	31.7	44.8	16.3	32.1
Bacillales	1.0	31.6	0.0	0.0	0.0	1.4	0.0	0.0	0.0	0.0	0.0	1.0	0.0	0.0	0.0	0.0	0.0	0.0	0.0
Enterobacteriales	1.0	1.0	2.2	2.4	1.4	0.0	0.0	0.0	0.0	0.0	0.0	0.0	0.0	0.0	0.0	0.0	0.0	0.0	0.0
Flavobacteriales	0.0	1.0	0.0	1.0	0.0	0.0	0.0	1.4	0.0	1.4	1.4	0.0	1.0	2.4	0.0	0.0	0.0	0.0	1.0
Micrococcales	1.4	0.0	0.0	0.0	0.0	0.0	0.0	0.0	0.0	0.0	0.0	0.0	0.0	0.0	0.0	0.0	0.0	0.0	0.0
Oceanospirillales	1.7	0.0	31.6	0.0	0.0	1.0	0.0	0.0	0.0	0.0	1.0	1.4	1.0	1.4	1.4	0.0	0.0	0.0	1.7
Pseudomonadales	5.5	5.0	7.8	8.0	4.6	30.7	1.4	7.5	5.2	5.3	5.6	8.8	4.5	6.4	7.4	3.6	4.0	5.0	4.0
Rhizobiales	0.0	0.0	0.0	0.0	0.0	0.0	0.0	0.0	0.0	0.0	0.0	0.0	0.0	0.0	1.0	0.0	0.0	0.0	0.0
Rhodobacterales	0.0	0.0	0.0	0.0	0.0	0.0	0.0	0.0	6.3	0.0	0.0	0.0	1.0	0.0	0.0	0.0	0.0	0.0	1.0
Vibrionales	5.5	44.9	2.2	2.4	2.6	31.8	31.6	2.4	0.0	12.5	0.0	1.0	2.4	31.8	3.0	1.4	1.7	2.6	1.0
Xanthomonadales	0.0	0.0	0.0	0.0	0.0	0.0	0.0	0.0	0.0	0.0	0.0	0.0	0.0	0.0	1.0	0.0	0.0	0.0	0.0
Fungi																			
Eurotiales	0.0	0.0	0.0	0.0	0.0	0.0	0.0	0.0	0.0	0.0	0.0	1.0	0.0	1.0	0.0	0.0	1.0	0.0	2.2
Hypocreales	0.0	0.0	0.0	0.0	0.0	0.0	0.0	0.0	0.0	0.0	0.0	0.0	0.0	0.0	0.0	0.0	0.0	1.0	0.0
Mucorales	0.0	0.0	0.0	1.4	0.0	0.0	0.0	0.0	0.0	0.0	0.0	0.0	0.0	0.0	0.0	0.0	0.0	0.0	0.0

Table S4. Analysis of RFLP band patterns of 19 *Mytilus* spp. individuals. "1": fragment present in the respective mussel individual, "0": fragment absent in the respective mussel individual.

**Individual**	**Fragment**					
	A (1100 bp)	B (950 bp)	C (850 bp)	D (700 bp)	E (600 bp)	F (550 bp)
1	0	1	0	0	1	0
2	0	1	0	0	0	1
3	0	1	0	0	1	0
4	0	1	0	0	1	0
5	0	1	0	0	1	0
6	0	1	1	0	0	1
7	0	1	0	0	1	0
8	0	1	1	0	1	1
9	0	1	0	0	1	0
10	0	0	1	0	0	1
11	0	1	0	0	1	1
12	1	0	0	0	1	0
13	0	0	1	0	0	1
14	0	1	0	0	1	0
15	0	1	0	0	1	0
16	0	1	0	0	1	0
17	0	1	1	0	0	1
18	0	0	0	1	0	1
19	0	0	1	0	1	0

Table S5. Putative identification of metabolites detected in farmed KF *Mytilus* species. Each detected compound is given with the experimentally determined *m/z* value (*m/z* exp.) and the *m/z* value calculated from the putative molecular formula (*m/z* calc.). Further, each putatively identified NP is given with the database(s) where the compound was found (DNP: Dictionary of Natural Products; UNPD: Universal Natural Products Database; CS: ChemSpider). [1]Putative molecular formulae were calculated by the elemental composition tool in the MassLynx software. IC: Identification confidence level after Sumner et al. 2007[1]. Nf: No fragmentation pattern detected. "-": No hit in databases used for the respective molecular formula.

Peak no.	*m/z* $[M+H]^+$ exp.	*m/z* $[M+H]^+$ calc.	RT (min)	Putative molecular formula[1]	IC	Fragmentation pattern	Putative identification	Data-base	Chemical family/class	Origin of NP	*Mytilus* sample	References
1	343.1709	343.1698	1.38	$C_{24}H_{22}O_2$	4	81.0689, 95.0859, 140.1430, 172.8558, 179.0481, 187.1454, 217.8574, 240.2292, 258.8575, 262.8551, 280.8629, 301.0711, 325.2066	-				7, 11, 12, 14	
2	375.1966	375.1556	1.38	$C_{19}H_{22}N_2O_6$	2	211.0757, 358.1908	3,3'-Methylenebistyrosine	DNP	Aminoacid derivative	*M. edulis*	7, 11, 12, 14	2
3	567.2571	567.2607	1.52	$C_{33}H_{35}N_4O_5$	4	507.2366, 549.2429	-				All	
4	734.4288	734.4281	1.66	$C_{44}H_{55}N_5O_5$	2	Nf	Aspochalamin C	UNPD	Alkaloid	*Aspergillus niveus*	All	3
5	283.2635	283.267	1.87	$C_{18}H_{35}O_2$	4	Nf	-				All	
6	326.377	326.3787	2.07	$C_{22}H_{47}N$	4	Nf	-				All	
7	311.2953	311.295	2.24	$C_{20}H_{38}O_2$	2	190.1158	a: Siphonarienolone, b: Iso-	DNP, UNPD	Polypropionate	*Siphonaria* sp.	All	a:[4], b:[5]
8	398.3763	398.3722	2.46	$C_{23}H_{47}N_3O_2$	4	Nf	-				8	
9	551.2629	551.4253	2.72	$C_{40}H_{54}O$	2	152.0548, 489.2212, 494.2249, 533.2465	Crocoxanthin	DNP, UNPD	Carotenoid	*M. edulis*	1-17	6
10	453.2966	453.2965	2.94	$C_{24}H_{40}N_2O_6$	4	280.2962	-				All	
11	467.3135	467.2121	3.02	$C_{25}H_{42}N_2O_6$	4	69.0689, 81.0712, 89.0589, 95.0847, 109.0986, 121.1012, 133.0834, 177.1062, 283.1688, 449.2840	-				All	
12	463.3007	463.3019	3.18	$C_{22}H_{42}N_2O_8$	4	89.0590, 111.0392, 127.0372, 133.0846, 142.0577, 186.0833, 199.0909, 215.0861, 243.1133, 283.1702, 337.1570, 357.1418, 377.1867	-				1-5, 7, 9, 11, 12, 15, 17	
13	455.3135	455.3121	3.28	$C_{24}H_{42}N_2O_6$	2	327.1976	Acremolide D	DNP, UNPD	Lipodepsipeptide	*Acremonium* sp.	All	7
14	467.3135	467.3134	3.32	$C_{26}H_{38}N_6O_2$	4	89.0587, 133.0844	-				All	

Peak no.	*m/z* [M+H]+ exp.	*m/z* [M+H]+ calc.	RT (min)	Putative molecular formula[1]	IC	Fragmentation pattern	Putative identification	Data-base	Chemical family/class	Origin of NP	*Mytilus* sample	References
15	503.2454	503.2447	3.41	$C_{32}H_{30}N_4O_2$	2	465.2940	Okaramine S	DNP	Indole diketopiperazine	*Aspergillus* sp.	All	8
16	565.2459	565.4046	3.41	$C_{40}H_{52}O_2$	3	420.2230, 441.7976, 477.2616, 492.2118, 503.2391, 521.2530, 537.2418	Alloxanthin analogue	DNP, UNPD	Carotenoid	*M. edulis*	All	6
17	579.2614	579.3838	3.41	$C_{40}H_{50}O_3$	2	Nf	Anhydroamarouciaxanthin B	DNP, UNPD	Carotenoid	*M. edulis*	All	6
18	597.2724	595.3787	3.41	$C_{40}H_{52}O_4$	2	477.2607, 503.2405, 521.2509,538.2520, 553.2772, 565.2405, 579.2562	Amarouciaxanthin B	DNP, UNPD	Carotenoid	*M. edulis* & other marine organisms	All	9
19	650.4911	650.4996	3.43	$C_{38}H_{67}NO_7$	2	184.0704	a: Pamamycin 649A, b: Pamamycin 649B	DNP, UNPD	Polyketide macrodiolide	*Streptomyces* sp.	All	10
20	595.2563	595.3787	3.58	$C_{40}H_{50}O_4$	2	503.3207, 535.3627, 553.2888	-				1-5, 7-16, 18, 19	
21	467.2743	467.3094	3.63	$C_{21}H_{38}N_8O_4$	4	341.2037	-				All	
22	553.2822	553.2815	3.68	$C_{32}H_{40}O_8$	2	89.0593, 133.0859, 521.2505	Tetronolide	DNP	Macrolide	*Micromonospora chalcea*	All	11
23	689.1858	689.1924	3.75	$C_{42}H_{28}N_2O_8$	4	117.0292, 309.2150, 373.1748, 391.1853, 403.2542, 451.2198, 467.2149, 531.1760, 625.2182	-				All	
24	578.4899	578.491	3.79	$C_{35}H_{59}N_7$	4	Nf	-				1, 4-19	
25	481.3254	481.3278	3.8	$C_{26}H_{44}N_2O_6$	2	89.0610, 104.1083, 133.0878, 177.1160, 283.1801, 308.3329, 355.3200, 416.3184, 443.2105	-				All	
26	469.2924	469.2927	3.91	$C_{25}H_{36}N_6O_3$	4	343.2197	-				All	
27	621.3896	621.3904	3.94	$C_{37}H_{52}N_2O_6$	4	Nf	-				All	
28	481.2884	481.2887	3.95	$C_{21}H_{36}N_8O_5$	4	89.0592, 133.0837, 283.1701, 355.2202	-				All	
29	703.2029	703.208	3.8-4.0	$C_{43}H_{30}N_2O_8$	4	117.0303, 180.9916, 323.2321, 387.1936, 389.2072, 405.2037, 417.2743, 465.2392, 481.2346, 545.1962, 639.2375	-				All	
30	741.4935	741.4934	4.04	$C_{44}H_{73}N_2O_2Br$	4	Nf	-				All	
31	639.3997	639.3969	4.04	$C_{32}H_{54}N_4O_9$	2	283.2589, 547.3308, 565.3189, 621.3820	Octacosamicin B	DNP, UNPD	Polyketide-PK-NRP	*Amycolatopsis azurea*	All	12

Peak no.	*m/z* [M+H]+ exp.	*m/z* [M+H]+ calc.	RT (min)	Putative molecular formula[1]	IC	Fragmentation pattern	Putative identification	Data-base	Chemical family/class	Origin of NP	*Mytilus* sample	References
32	717.2143	717.2183	4.06	$C_{38}H_{36}O_{14}$	4	117.0292, 180.9903, 337.2455, 401.2061, 419.2172, 495.2467, 559.2080, 653.2491	-				All	
33	585.2461	585.2448	4.11	$C_{30}H_{36}N_2O_{10}$	2	Nf	Renieramycin P	DNP	Tetrahydro-isoquinoline alkaloid	*Neopetrosia* sp.	2, 4-12, 14-16, 19	13
34	621.3898	621.3944	4.2	$C_{37}H_{52}N_2O_6$	4	269.2440, 283.2595, 471.2797, 529.3324, 533.2115, 535.3484, 545.1949, 563.3427	-				All	
35	581.2424	581.3995	4.29	$C_{40}H_{52}O_3$	2	89.0591, 97.0984, 105.0699, 109.0998, 117.0297, 133.0842, 221.1329, 239.0484, 255.2284, 282.2765, 295.1040, 311.2855, 323.2285, 329.2988, 337.0180, 371.2201, 387.1882, 393.0819, 413.0503, 423.1714, 469.1112, 481.2310, 525.1714, 545.1940	4-Hydroxy-alloxanthin (Variant 3*S*,3'*R*,4*R*- or 3*S*,3'*R*,4*S*-form)	DNP	Carotenoid	*M. coruscus*	All	14
36	593.274	593.3631	4.37	$C_{40}H_{48}O_4$	2	117.0299, 401.2057, 533.2469, 559.2086	7,7',8,8'-Tetradehydro-astaxanthin	DNP, UNPD	Carotenoid	*M. coruscus*	All	15
37	389.2121	389.2117	4.41	$C_{26}H_{29}O_3$	4	Nf	-				All	
38	547.2162	547.2179	4.41	$C_{28}H_{34}O_{11}$	2	117.0295, 180.9903, 325.2456, 389.2072, 483.2469	3-*O*-α-L-Rhamnopyranosyl-α-galactose	DNP, UNPD	Polysaccharide	*Salmonella* sp.	All	16
39	567.2967	567.2971	4.41	$C_{34}H_{38}N_4O_4$	2	Nf	Corallistin B	DNP, UNPD	Porphyrin	*Corallistes* sp.	All	17
40	587.2286	587.2281	4.47	$C_{34}H_{34}O_9$	4	117.0296, 389.2067, 483.2108, 511.2057, 527.2001, 543.2313, 547.2081	-				All	
41	589.244	589.2438	4.47	$C_{34}H_{36}O_9$	4	117.0305, 389.2087, 483.2145, 511.2088, 513.2234, 527.2038, 543.2353, 547.2120	-				All	
42	531.2356	531.2343	4.47	$C_{27}H_{34}N_2O_9$	4	515.2372	-				All	
43	533.2552	533.2553	4.59	$C_{33}H_{32}N_4O_3$	2	461.2652, 474.2371, 505.2548, 515.2399	Chlorophyllone a	DNP, UNPD	Tetrapyrrole	*Crassostrea* sp., *Patinopecten yessoensis, Ruditapes*	All	18

Peak no.	*m/z* [M+H]$^+$ exp.	*m/z* [M+H]$^+$ calc.	RT (min)	Putative molecular formula[1]	IC	Fragmentation pattern	Putative identification	Data-base	Chemical family/class	Origin of NP	*Mytilus* sample	References
										philippinarum		
44	535.2703	535.2709	4.68	$C_{33}H_{34}N_4O_3$	2	507.2710	Pyropheophorbide a	DNP, UNPD	Tetrapyrrole	*Pterocladiatennis*, *Ruditapes philippinarum*, *Trididemnum solidum*	All	19
45	565.2457	565.4046	4.75	$C_{40}H_{52}O_2$	3	420.2276, 475.2453, 477.2602, 492.2124, 493.2478, 503.2410, 521.2512, 537.2457	Alloxanthin analogue	DNP, UNPD	Carotenoid	*M. edulis*	All	6
46	521.2553	521.255	4.75	$C_{32}H_{32}N_4O_3$	2	477.2610, 493.2548, 503.2400	a: Okaramine A, b: Okaramine H	DNP, UNPD	Indole diketopiperazine	*Penicillium simplicis-simum*, H also from *Aspergillus aculeatus*	All	a:[20], b:[21]
47	531.2356	531.2383	4.94	$C_{32}H_{34}O_7$	4	458.2391	-				All	
48	621.3923	621.3944	4.94	$C_{35}H_{56}O_9$	2	529.3249	9-Hydroxybafilomycin D	CS	Macrolide	*Streptomyces* sp.	All	22
49	621.3931	621.3944	5	$C_{35}H_{56}O_9$	2	529.3254	9-Hydroxybafilomycin D	CS	Macrolide	*Streptomyces* sp.	All	22
50	565.2809	565.4046	5.08	$C_{40}H_{52}O_2$	2	547.2620	Alloxanthin		Carotenoid	*M. edulis*	1-12, 14, 16	6
51	519.2744	519.276	5.27	$C_{33}H_{34}N_4O_2$	2	Nf	$13^2,17^3$-cyclomesopheophorbide-*a*-enol	DNP	Tetrapyrrole	Marine sediments (derivative in bivalves and sponges)	All	23,24
52	503.2435	503.2434	5.49	$C_{31}H_{34}O_6$	4	268.2971	-				All	
53	531.244	531.2383	5.49	$C_{32}H_{34}O_7$	4	Nf	-				All	
54	585.2472	585.2488	5.49	$C_{35}H_{36}O_8$	2	Nf	Antibiotic C 104	DNP	Angucyclines	*Streptomyces* sp.	All	25
55	633.1509	633.4155	5.59	$C_{40}H_{56}O_6$	2	Nf	Fucoxanthinol	UNPD	Carotenoid	*M. edulis*	All	6
56	355.0703	355.0695	5.59	$C_{19}H_{11}N_2O_4$	4	Nf	-				All	
57	547.2711	547.2696	5.83	$C_{33}H_{38}O_7$	4	Nf	-				All	
58	748.5336	748.5323	5.94	$C_{38}H_{73}N_3O_{11}$	4	Nf	-				All	
59	563.2642	563.2645	5.99	$C_{33}H_{38}O_8$	4	504.2436, 532.2392, 235.2632	-				1-15, 18, 19	
60	397.2346	397.347	6.23	$C_{28}H_{44}O$	2	Nf	Ergosterol	DNP, UNPD	Sterol	*M. edulis*	All	26
61	732.5358	732.5374	6.33	$C_{38}H_{73}N_3O_{10}$	4	184.0715	-				All	
62	762.5453	762.5421	6.46	$C_{46}H_{71}N_3O_6$	4	Nf	-				All	
63	707.1696		6.89	$C_{30}H_{34}N_4O_{12}S_2$	4	Nf	-				All	
64	642.4832	642.4806	7.19	$C_{33}H_{63}N_5O_7$	2	Nf	Procidin S 735	DNP	Peptide	*Streptomyces procidinanus*	All	27

Peak no.	*m/z* [M+H]+ exp.	*m/z* [M+H]+ calc.	RT (min)	Putative molecular formula[1]	IC	Fragmentation pattern	Putative identification	Data-base	Chemical family/class	Origin of NP	*Mytilus* sample	References
65	586.4803	586.4795	7.35	$C_{32}H_{63}N_3O_6$	4	Nf	-				All	
66	556.4675	566.4689	7.77	$C_{31}H_{61}N_3O_5$	4	89.0587, 133.0863	-				All	
67	647.1643	647.1612	7.89	$C_{30}H_{30}O_{16}$	4	Nf	-				All	
68	355.0691	355.0695	7.9	$C_{19}H_{11}N_2O_4$	4	Nf	-				All	
69	653.4509	653.4489	7.99	$C_{34}H_{60}N_4O_8$	2	325.2089, 351.2228	-				All	
70	815.5648	815.5622	8.25	$C_{48}H_{78}O_{10}$	2	Nf	Halstoctacosanolide C	DNP, UNPD	Polyketide macrolide	*Streptomyces halstedii*	All	28
71	570.4854	570.4862	8.29	$C_{35}H_{64}NO_3$	4	89.0603, 133.0836, 552.4629	-				All	
72	781.1877	781.1836	8.45	$C_{48}H_{24}N_6O_6$	4	Nf	-				All	
73	709.4783	709.4792	8.87	$C_{42}H_{64}N_2O_7$	4	407.2477	-				1, 3, 4, 6, 8-13, 15-17	
74	817.58	817.579	9.31	$C_{43}H_{80}N_2O_{12}$	4	Nf	-				All	
75	721.1829	721.1822	9.64	$C_{42}H_{28}N_2O_{10}$	4	Nf	-				All	
76	855.2072	855.2136	10.1	$C_{44}H_{38}O_{18}$	4	Nf	-				All	

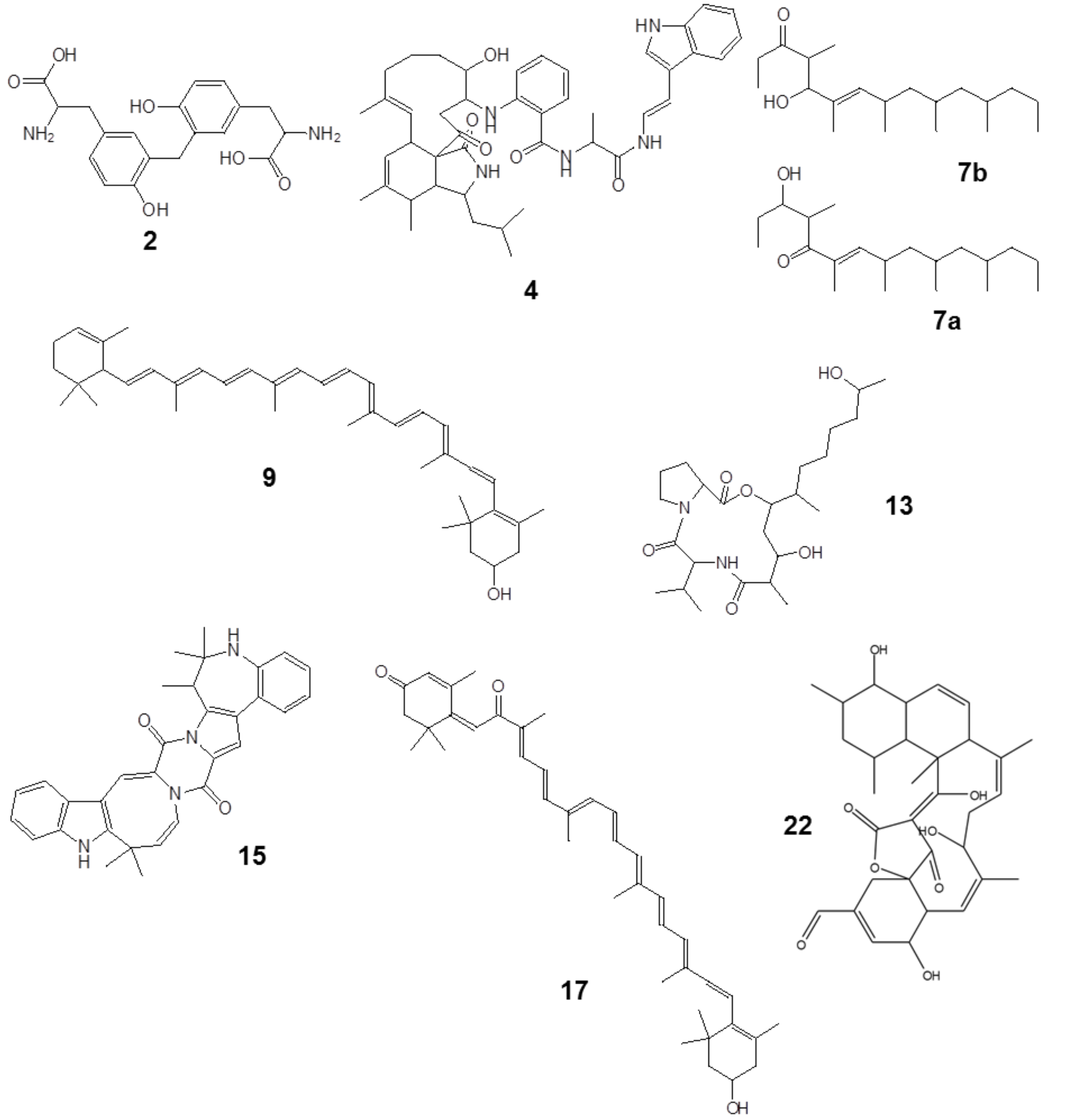

Figure S1. Chemical structures of identified compounds in extracts of blue mussels by UPLC-QTOF-MS/MS analysis. Chemical structures are given with the respective peak number. Detailed information (*i.e.* identity, characteristic MS data and references) for of all compounds presented here can be found in the supplementary material (Supplementary Table S5). Chemical structures of the following compounds can be found in Fig. 5: **18, 19a**, **19b**, **43**, **46a**, **46b, 60**.

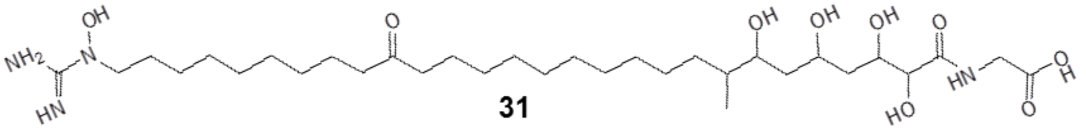

31

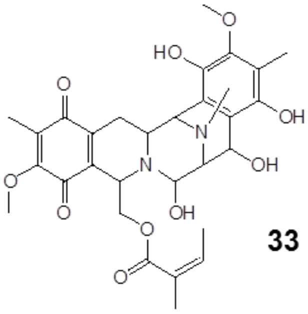

33

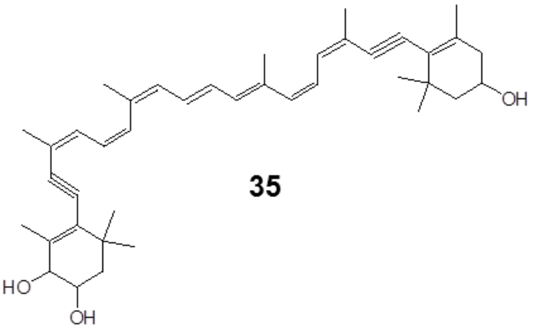

35

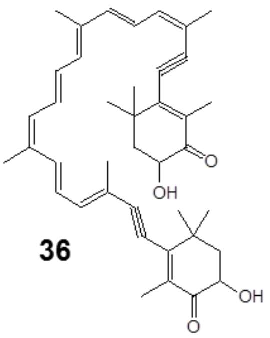

36

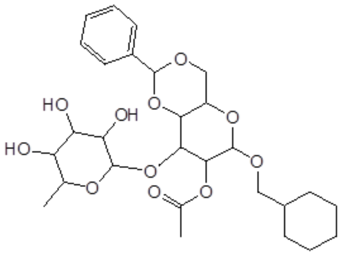

38

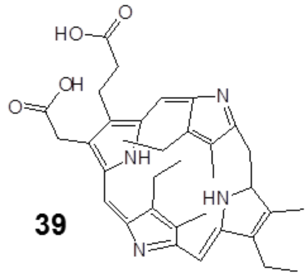

39

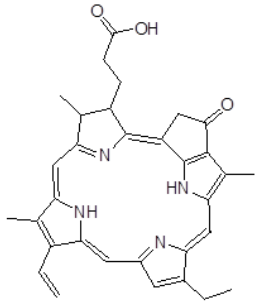

44

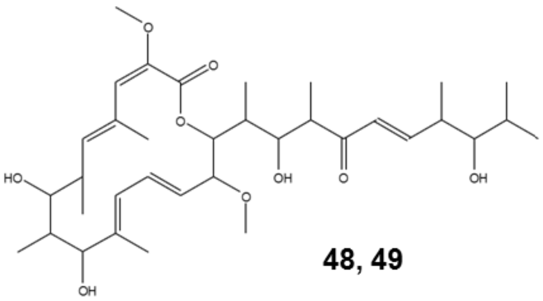

48, 49

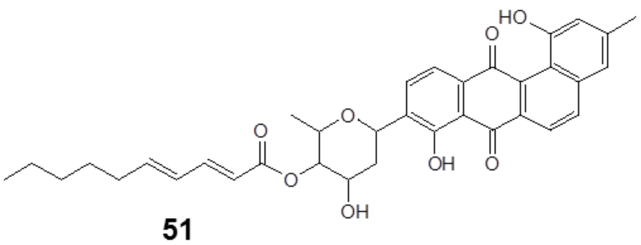

51

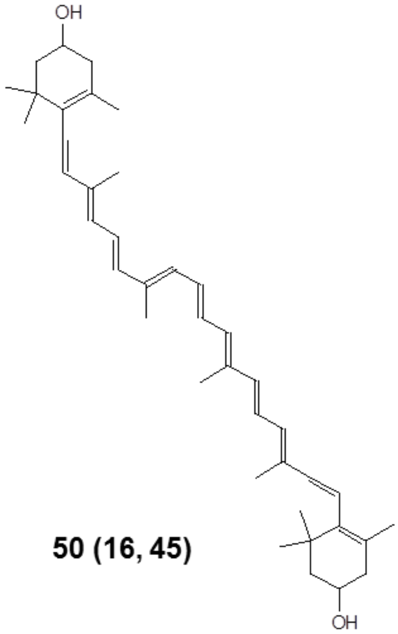

50 (16, 45)

S1 Fig. (continued)

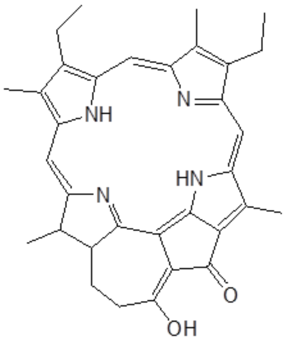

54

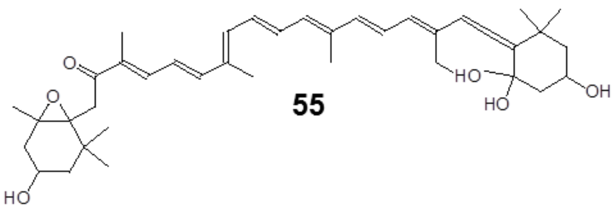

55

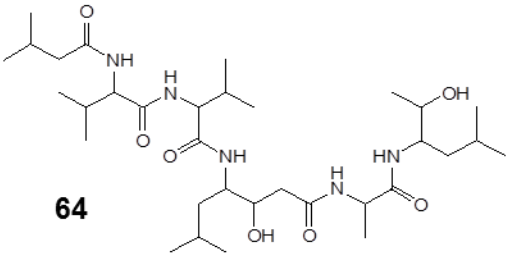

64

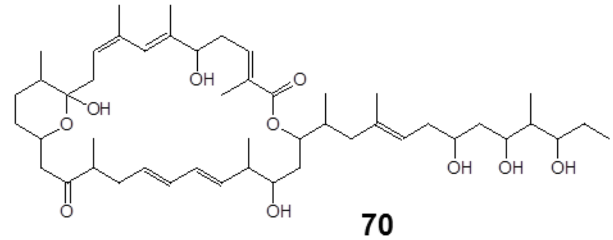

70

S1 Fig. (continued)

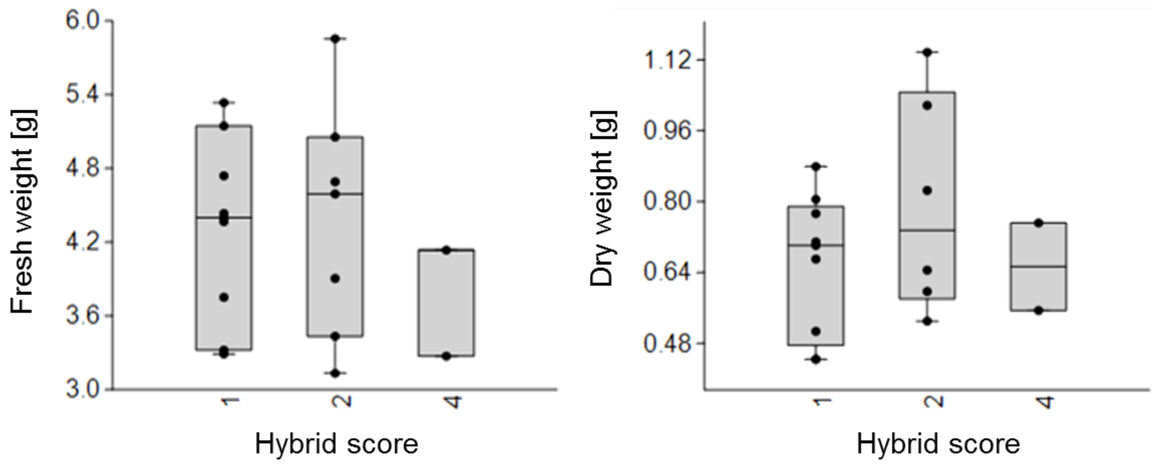

Figure S2. Distribution of fresh and dry weight among the different genotypes (n=18). Data are presented as box-whisker plots (mean ± SD) sorted by genotype (Hybrid scores: 1: *M. trossulus*-like hybrid, 2: intermediate hybrid, 4: pure *M. edulis*).

Additional references for supplementary information (accompany Table S5).

1 Sumner, L. W. *et al.* Proposed minimum reporting standards for chemical analysis Chemical Analysis Working Group (CAWG) Metabolomics Standards Initiative (MSI). *Metabolomics* **3**, 211-221 (2007).

2 Andersen, S. O. Isolation of a new type of cross link from the hinge ligament protein of molluscs. *Nature* **216**, 1029-1030 (1967).

3 Gebhardt, K. *et al.* Aspochalamins A-D and aspochalasin Z produced by the endosymbiotic fungus *Aspergillus niveus* LU 9575. *J. Antibiot.* **57**, 707-714 (2004).

4 Norte, M., Cataldo, F. & González, A. G. Siphonarienedione and siphonarienolone, two new metabolites from *Siphonaria grisea* having a polypropionate skeleton. *Tetrahedron Lett.* **29**, 2879-2880 (1988).

5 Paul, M. C., Zubia, E., Ortega, M. J. & Salva, J. New polypropionates from *Siphonaria pectinata*. *Tetrahedron* **53**, 2303-2308 (1997).

6 Hertzberg, S., Partali, V. & Liaaen-Jensen, S. Animal carotenoids. 32. Carotenoids of *Mytilus edulis* (edible mussel). *Acta Chem. Scand. B* **42**, 495-503 (1988).

7 Ratnayake, R., Fremlin, L. J., Lacey, E., Gill, J. H. & Capon, R. J. Acremolides A-D, lipodepsipeptides from an Australian marine-derived fungus, *Acremonium* sp. *J. Nat. Prod.* **71**, 403-408 (2008).

8 Cai, S. *et al.* Okaramines S–U, three new indole diketopiperazine alkaloids from *Aspergillus taichungensis* ZHN-7-07. *Tetrahedron* **71**, 3715-3719 (2015).

9 Partali, V., Tangen, K. & Liaaen-Jensen, S. Carotenoids in food chain studies—III. Resorption and metabolic transformation of carotenoids in *Mytilus edulis* (edible mussel). *Comp. Biochem. Physiol. B* **92**, 239-246 (1989).

10 Natsume, M., Yasui, K., Kondo, S. & Marumo, S. The structures of four new pamamycin homologues isolated from *Streptomyces alboniger*. *Tetrahedron Lett.* **32**, 3087-3090 (1991).

11 Takeda, K. *et al.* Studies on the synthesis of tetronolide. Synthesis of a spiro-a-acyltetronic acid model. *J. Org. Chem.* **50**, 4673-4681 (1985).

12 Dobashi, K., Naganawa, H., Takahashi, Y., Takita, T. & Takeuchi, T. Novel antifungal antibiotics octacosamicins A and B. II. The structure elucidation using various NMR spectroscopic methods. *J. Antibiot.* **41**, 1533-1541 (1988).

13 Oku, N., Matsunaga, S., van Soest, R. W. & Fusetani, N. Renieramycin J, a highly cytotoxic tetrahydroisoquinoline alkaloid, from a marine sponge *Neopetrosia* sp. *J. Nat. Prod.* **66**, 1136-1139 (2003).

14 Maoka, T. & Matsuno, T. Isolation and structural elucidation of three new acetylenic carotenoids from the Japanese sea mussel *Mytilus coruscus*. *Nippon Suisan Gakk* **54**, 1443-1447 (1988).

15 Maoka, T. A new apocarotenoid from the marine shellfish *Mytilus coruscus*. *J. Nat. Prod.* **60**, 616-617 (1997).

16 Szurmai, Z. & Lipták, A. Synthesis and ^{13}C-NMR spectroscopy of 2-*O*-and 6-*O*-acetyl-3-*O*-α-L-rhamnopyranosyl-D-galactose, constituents of bacterial cell-wall polysaccharides. *Carbohydr. Res.* **107**, 33-41 (1982).

17 D'Ambrosio, M., Guerriero, A., Pietra, F., Debitus, C. & Ribes, O. On the novel free porphyrins Corallistin B, C, D, and E: Isolation from the demosponge *Corallistes* sp. of the Coral Sea and reactivity of their Nickel 55 (II) complexes toward formylating reagents. *Helv. Chim. Acta* **76**, 1489-1496 (1993).

18 Sakata, K. *et al.* Chlorophyllone-a, a new pheophorbide-a related compound isolated from *Ruditapes philippinarum* as an antioxidative compound. *Tetrahedron Lett.* **31**, 1165-1168 (1990).

19 Watanabe, N. *et al.* New chlorophyll-a-related compounds isolated as antioxidants from marine bivalves. *J. Nat. Prod.* **56**, 305-317 (1993).

20 Murao, S., Hayashi, H., Takiuchi, K. & Arai, M. Okaramine A, a novel indole alkaloid with insecticidal activity, from *Penicillium simplicissimum* Ak-40. *Agr Biol Chem Tokyo* **52**, 885-886 (1988).

21 Hayashi, H., Furutsuka, K. & Shiono, Y. Okaramines H and I, new okaramine congeners, from *Aspergillus aculeatus*. *J. Nat. Prod.* **62**, 315-317 (1999).
22 Yu, Z. *et al.* Bafilomycins produced by an endophytic actinomycete *Streptomyces* sp. YIM56209. *J. Antibiot.* **64**, 159-162 (2011).
23 Louda, J. W., Loitz, J. W., Rudnick, D. T. & Baker, E. W. Early diagenetic alteration of chlorophyll-a and bacteriochlorophyll-a in a contemporaneous marl ecosystem; Florida Bay. *Org. Geochem.* **31**, 1561-1580 (2000).
24 Khalesi, M. & Louda, J. W. Hemisynthesis of 13^2,17^3-cyclomesopheophorbide-*a*-enol. *Tetrahedron Lett.* **52**, 1078-1081 (2011).
25 Matsumoto, T., Sohma, T., Yamaguchi, H., Kurata, S. & Suzuki, K. Total synthesis of antibiotic C104: Benzyne-Furan cycloaddition approach to the angucyclines. *Tetrahedron* **51**, 7347-7360 (1995).
26 van der Vliet, J. Investigations on sterols III: The Provitamins-D from the mussel *Mytilus edulis*. *Recl. Trav. Chim. Pays-Bas* **67**, 265-281 (1948).
27 Kojima, S., Koide, T., Ogino, S., Tsuchiya, T. & Kameno, Y. Protease inhibitors. US 4091093 A (1978).
28 Tohyama, S., Kakinuma, K. & Eguchi, T. The complete biosynthetic gene cluster of the 28-membered polyketide macrolactones, halstoctacosanolides, from *Streptomyces halstedii* HC34. *J. Antibiot.* **59**, 44-52 (2006).
